# Transcriptomic Analysis of Cadmium Stress Response in the Heavy Metal Hyperaccumulator *Sedum alfredii* Hance

**DOI:** 10.1371/journal.pone.0064643

**Published:** 2013-06-03

**Authors:** Jun Gao, Ling Sun, Xiaoe Yang, Jian-Xiang Liu

**Affiliations:** 1 MOE Key Laboratory of Environment Remediation and Ecological Health, College of Natural Resource & Environmental Sciences, Zhejiang University, Hangzhou, China; 2 State Key Laboratory of Genetic Engineering, Institute of Plant Biology, School of Life Sciences, Fudan University, Shanghai, China; University of Michigan, United States of America

## Abstract

The *Sedum alfredii* Hance hyperaccumulating ecotype (HE) has the ability to hyperaccumulate cadmium (Cd), as well as zinc (Zn) and lead (Pb) in above-ground tissues. Although many physiological studies have been conducted with these plants, the molecular mechanisms underlying their hyper-tolerance to heavy metals are largely unknown. Here we report on the generation of 9.4 gigabases of adaptor-trimmed raw sequences and the assembly of 57,162 transcript contigs in *S. alfredii* Hance (HE) shoots by the combination of Roche 454 and Illumina/Solexa deep sequencing technologies. We also have functionally annotated the transcriptome and analyzed the transcriptome changes upon Cd hyperaccumulation in *S. alfredii* Hance (HE) shoots. There are 110 contigs and 123 contigs that were up-regulated (Fold Change ≧2.0) and down-regulated (Fold Change ≦0.5) by chronic Cd treatment in *S. alfredii* Hance (HE) at q-value cutoff of 0.005, respectively. Quantitative RT-PCR was employed to compare gene expression patterns between *S. alfredii* Hance (HE) and non-hyperaccumulating ecotype (NHE). Our results demonstrated that several genes involved in cell wall modification, metal translocation and remobilization were more induced or constitutively expressed at higher levels in HE shoots than that in NHE shoots in response to Cd exposure. Together, our study provides large-scale expressed sequence information and genome-wide transcriptome profiling of Cd responses in *S. alfredii* Hance (HE) shoots.

## Introduction

The non-essential heavy metal cadmium (Cd) in soils is very mobile and readily enters plant tissues, the food chain and drinking water. The major concern about Cd pollution is its potential threat to human health with the high risk of causing cancer including lung, bladder, renal, prostate, and breast cancer [1], [2]. Cd interferes with cell proliferation, differentiation, DNA replication and repair, as well as protein synthesis and folding [2]. Cd is also toxic to plant tissues, adversely affecting the photosynthetic apparatus, carbohydrate metabolism and nitrate absorption [3]. Tolerant plants differ in the ways in which they contend with Cd. “Excluders” tolerate heavy metals by limiting the entry and root-to-shoot translocation of trace metals. On the other hand, hyper-accumulating plants can accumulate extremely high levels of heavy metals in their above-ground tissues without exhibiting toxicity symptoms. Hyper-accumulating plants show promise for use in phytomediation, i.e., for cleaning up heavy metal contaminated sites and reducing the bioavailability of heavy metals in the environment [4]. However, the use of hyperaccumulators for this purpose has been limited due to their low biomass, lack of metal selectivity or poor agronomic practice. Understanding the molecular mechanisms of hyperaccumulation may help in enhancing the performance of hyperaccumulators for phytoremediation [5].

So far over 450 heavy metal accumulators have been identified, most of which (75%) are nickel (Ni) tolerant [6]. Cd hyperaccumulators have been defined as plants that accumulate and tolerate of at least 100 mg kg^−1^ dry weight (DW) in shoots of plants [6]. Cd hyperaccumulation occurs in four species of *Brassicaceae* and *Crassulaceae* family. *Thlaspi caerulescens*, also known as *Noccaea caerulescens*, and *Arabidopsis halleri* are two of the best studied hyperaccumulating species in *Brassicaceae* family, and the availability of *Arabidopsis thaliana* genome sequence information has helped in identify genes associated with heavy metal tolerance in its close relatives, *T. caerulescens* and *A. halleri* [7], [8]. *A. halleri* has been found to have a high constitutive level of Zn/Cd transporter *HMA4* (P-type ATPase) expression apparently due to a modification in *cis*-regulatory sequences and gene copy number expansion [9]. In other species, heavy metal hyper-tolerance is mainly associated with vacuolar sequestration and metal chelation, both of which are heavy metal inducible [10]. Recently, the constitutive, high-level expression of a tonoplast-localized transporter HMA3 (ATPase) was reported to be associated with specific Cd sequestration into vacuoles in *T. caerulescens* leaves [11].

*Sedum alfredii* Hance hyperaccumulating ecotype (HE) is the only hyperaccumulating species in *Crassulaceae* family identified to date, originally found as a Zn hyperaccumulator in a soil-Pb/Zn-rich region in China [12]. It is not only a Zn/Cd cohyperaccumulator, but also highly tolerant to copper (Cu) and Pb toxicity [13], [14], [15]. A number of physiological studies have been carried out to understand the hyper-tolerance and hyperaccumulation mechanisms in *S. alfredii* Hance (HE) [16], [17], [18], [19]. In contrast to *T. caerulescens* and *A. halleri,* the molecular basis for hyper-tolerance in *S. alfredii* Hance (HE) is not known, due in part to our lack knowledge about the genome of this organism.

The development of high-throughput deep sequencing technology has enabled the large-scale RNA-seq of dynamic transcriptomes, even without fully sequenced reference genomes [20], [21]. In the present study, we have employed RNA-seq to explore the transcriptome of *S. alfredii* Hance (HE) and to identify transcriptional changes in response to high Cd accumulation in shoots. We also compared the expression levels of selected genes in shoots of *Sedum alfredii* Hance (HE) and the non-hyperaccumulating ecotype (NHE). We observed that several genes involved in cell wall modification and metal translocation were more induced by Cd or constitutively expressed at higher levels under normal growth condition in HE than that in NHE. Our data will help in furthering our understanding of the molecular mechanisms of heavy metal hyper-tolerance in plants.

## Results

### Investigation the Transcriptome of *S. alfredii* Hance (HE) Shoots

**Figure 1:**
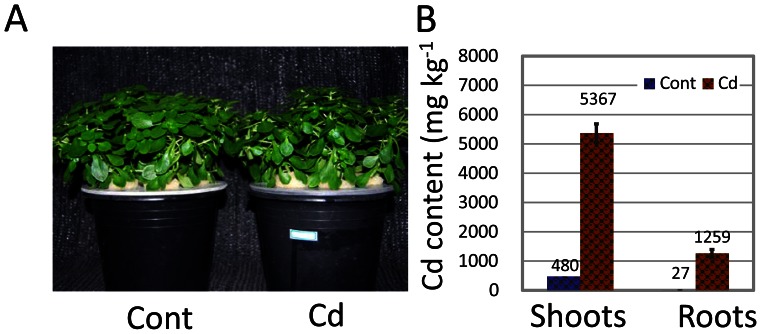
Cd hyperaccumulation in *S. alfredii* Hance (HE). (A) Growth of HE after exposure to 0 µM (Cont) or 100 µM Cd^2+^ (Cd) for 8 days. (B) Cd content (mg kg^−1^ dry weight) in shoots and roots of HE. Bars depict SE (n = 3).

*S. alfredii* Hance (HE) can accumulate more than 5,000 mg kg^−1^ DW of Cd in shoots without any observable symptoms of toxicity when grown hydroponically (Figure 1A–B). *S. alfredii* Hance (HE) shoots were used in most of these studies since Cd content in shoots was much higher than that in roots (Figure 1B), and high-quality RNA was better obtained from shoots than roots in this species. Initially, RNAs from Cd treated (Cd) or untreated control (Cont) samples were pooled and sequenced on the Roche’s 454 sequencing platform. Pooling of the sequencing runs of Cd and Cont samples resulted in 135,894 qualified reads with an average length of 287 bases (b) after removal of adaptor sequences (Figure S1A), which were equal to 39.1 millionbases (Mb) in total. Subsequently, Illumina/Solexa cDNA libraries were constructed and sequenced from Cd and Cont samples with three biological replicates, respectively. In total, around 9.4 gigabases (Gb) of adaptor-trimmed raw sequences representing 47.5 million qualified reads with an average of 99 b were obtained from Illumina/Solexa sequencing (Table S1). The 454 reads were firstly assembled with Newbler to generate 4,193 EST clusters (isotigs) containing 83,333 reads (61.3% of total), which provided a reference framework for the later assembly of Illumina/Solexa sequences. First, 47,504,847 Illumina/Solexa reads (22.9% of total) were mapped to 454-derived isotigs; the rest of the Illumina/Solexa reads were separately assembled using Velvet/Oases [22] together with the 454 singleton reads, yielding 119,731 EST clusters (contigs). The aforementioned isotigs and contigs were finally re-assembled in CAP3 [23] to generate the transcriptome of *S. alfredii* Hance (HE) shoots which contains 57,162 contigs with lengths longer than 200 b (Figure S1B).

### Functional Annotation of the *S. alfredii* Hance (HE) Transcriptome

**Figure 2:**
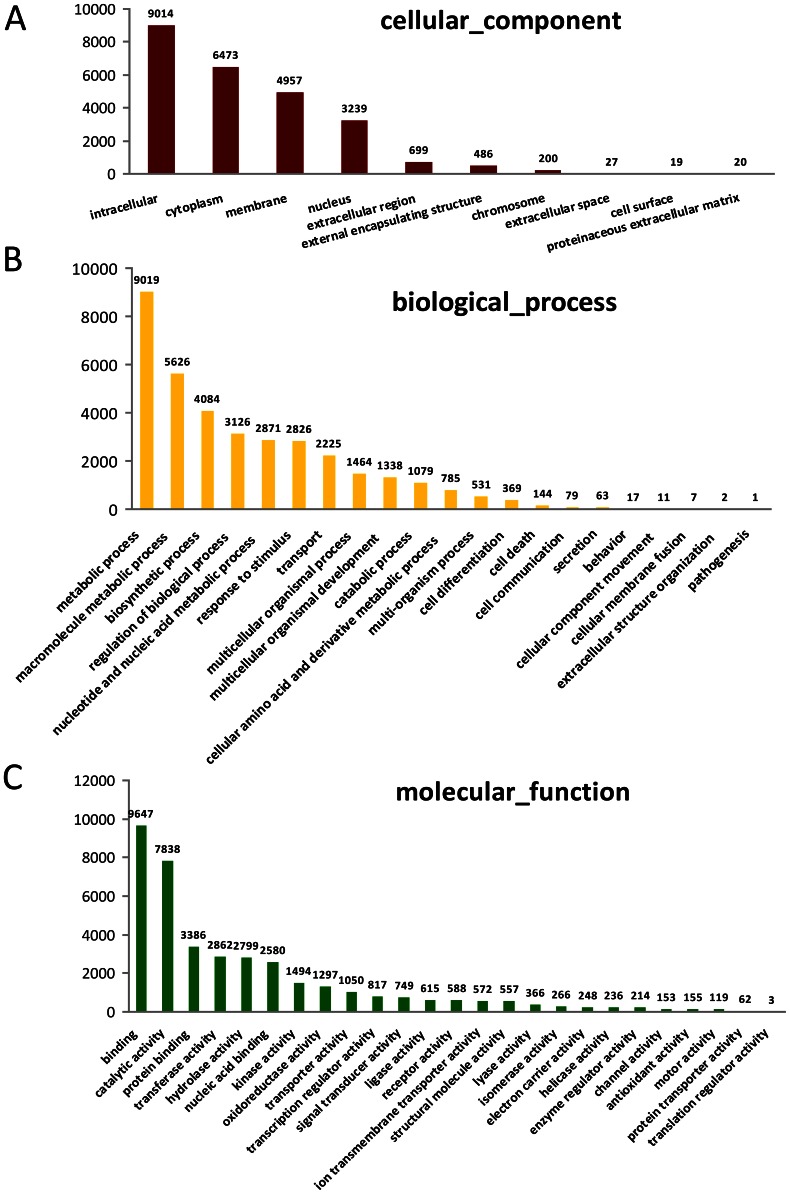
Gene Ontology (GO) analysis of *S. alfredii* Hance (HE) contigs. GO distribution of (A) cellular component, (B) biological process and (C) molecular function.

**Figure 3:**
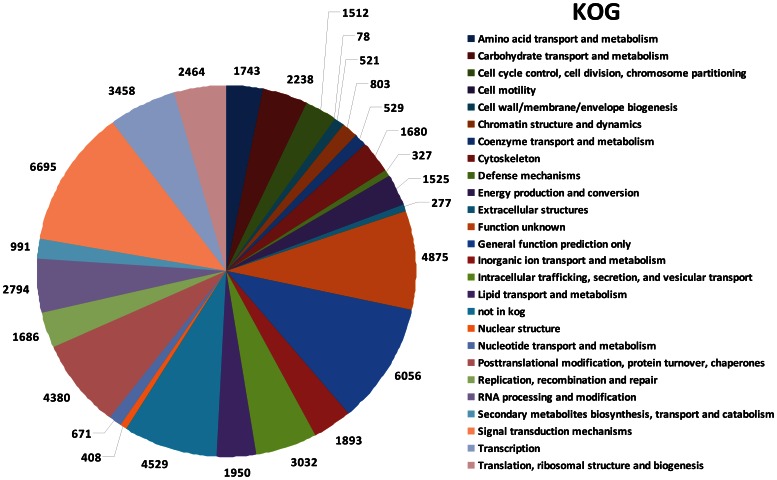
Proportion of *S. alfredii* Hance (HE) contigs in different functional categories. KEGG (Kyoto Encyclopedia of Genes and Genomes) Orthology Groups (KOG) analysis was applied to the assembled contigs.

**Figure 4:**
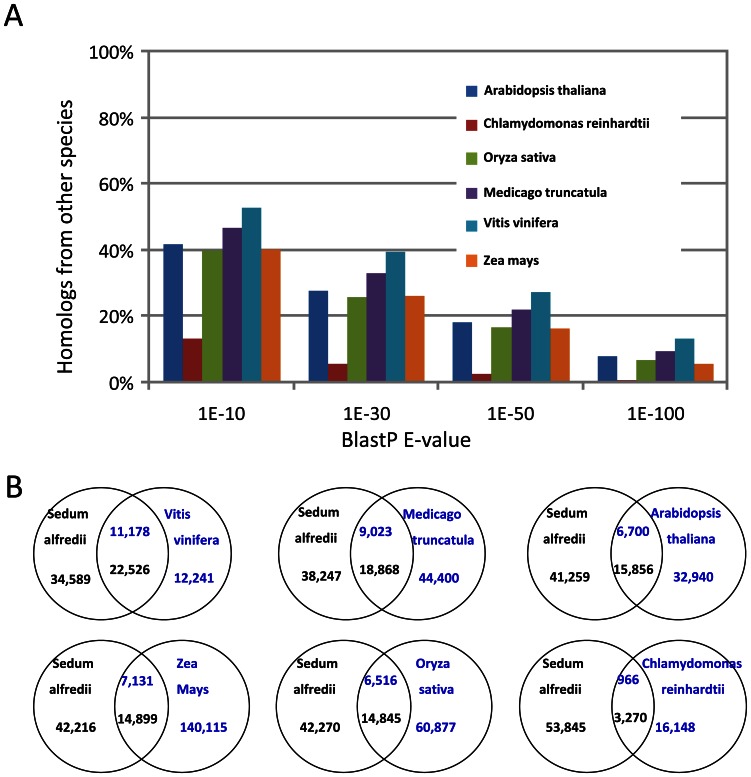
Homologous protein comparisons of *S. alfredii* Hance (HE) to other sequenced model plants. (A) Distribution of *S*. *alfredii* homologous proteins identified in other plants using several BlastP E-value cutoffs. (B) Comparison of the number of homologs identified between *S*. *alfredii* and other plant proteomes with BlastP E-value cutoff of 1E-30. The number of contigs in each species is displayed in the same color as the species name.

Gene prediction was carried out with the GETORF program using the EMBOSS package [24] and 57,115 protein-coding contigs were identified. All the predicted proteins were compared with the non redundant protein sequence databases in Swiss-Prot and GeneBank using BlastP searches. A total of 28,578 contigs were functionally annotated with an E-value cut-off of 1E-3. Gene Ontology (GO) analysis was subsequently performed with GoPipe [25]. In total, 14,046 contigs were matched to 75,775 GO terms (Figure 2A–C). The largest cellular component for *S. alfredii* Hance (HE) proteome represented intracellular components (35.9%, Figure 2A). The majority of biological processes identified were involved in metabolic processes (25.3%, Figure 2B) and macromolecule metabolic processes (15.8%, Figure 2B). Most of the molecular functions were associated with binding (25.0%, Figure 2C) and catalytic activities (20.3%, Figure 2C). The largest group in KEGG Orthology (KO, Figure 3) was related to signal transduction mechanisms (11.7%), and the smallest group was cell motility (0.1%). In total, 4,380 of the contigs (7.7%) were associated with posttranslational modification, protein turnover and chaperones while 3458 contigs (6.1%) were related to transcription. Totally 649 contigs encoding putative transcription factors were identified; among them, zinc finger proteins (16.3%) and MYB (16.2%) were the most abundant followed by bHLH (12.5%) and bZIP transcription factors (8.3%). Pathway enrichment of the transcriptome was also conducted according to GO annotations for KEGG. 5,023 contigs were found to be involved in 37 different pathways (Figure S2). When compared to protein databases from six sequenced model plant species (*Chlamydomonas reinhardtii*, *Arabidopsis thaliana*, *Oryza sativa*, *Zea mays*, *Medicago truncatula* and *Vitis vinifera*), the *S. alfredii* Hance (HE) proteome was most similar to the *V. vinifera* proteome, followed by the *M. truncatula* proteome (Figure 4A–B), both of which are in dicotyledous plants in the same *Rosidae* subclass as *S. alfredii* Hance. Given a BlastP E-value cutoff of 1E-30, 47.7% of *V. vinifera* proteins were found to be homologous to 39.4% of *S. alfredii* Hance proteins/contigs, whereas 16.9% of *M. truncatula* proteins were similar to 33.0% of *S. alfredii* Hance proteins/contigs. The green alga *C. reinhardtii* contigs had the lowest overall similarity to the *S. alfredii* Hance contigs (Figure 4B). Microsatellites, also known as simple sequence repeats (SSRs), are widely recognized as useful co-dominant, locus-specific markers for DNA fingerprinting, genome mapping and phylogenetic analysis [26]. Among the 57,162 contigs in *S. alfredii* Hance (HE), 6,176 perfect SSRs and 3,019 imperfect SSRs were found (Dataset S1). Many species in *Crassulaceae* family are CAM plants, in which water use efficiency is increased and malic acid level is elevated [27]. Phosphoenolpyruvate carboxylase (PEPC) is a key enzyme in CAM and C4 metabolism and has been used as a marker for phylogenetic classification among C3, C4 and CAM plants [28]. Twenty contigs encoding PEPC homologs were found in the *S. alfredii* Hance (HE) transcriptome (Table S3) and phylogenetic analysis revealed that *S. alfredii* Hance (HE) had both C3- and CAM-type PEPCs. Among the *S. alfredii* Hance PEPCs, one contig (*Sa_Contig08207*) was most closely related to CAM-type PEPC in *K. blossfeldiana*, which is also in the *Crassulaceae* family (Figures S3–S4). These results indicate that *S. alfredii* Hance (HE) are CAM plants, and our deep sequencing data may be useful for CAM studies in the future.

### Comparative Transcriptome Analysis in *S. alfredii* Hance (HE) Shoots

Long exposure of *S. alfredii* Hance (HE) to Cd was employed in the present study to investigate the molecular mechanisms of Cd hyper-tolerance to minimize the interference of acute phase responses. The expression level of each contig was calculated and normalized to RPKM (Reads Per Kilobase of exon model per Million total reads in sample) according to standard protocols [29]. Compared with the Cont, totally 110 contigs were upregulated and 123 contigs were down-regulated by Cd treatment (FC≧2.0 and FC≦0.5, respectively) at a q-value cutoff of 0.005 (Table S2). The upregulated contigs were grouped into five categories according to their functional annotations (Table 1). One category included 20 contigs, which were related to phenylpropanoid biosynthesis, cell wall deposition and modification. Another category included 4 contigs for heavy metal efflux and 7 contigs related to metal ligand synthesis and metal-ligand transport. There were 6 contigs (2 peroxiredoxins and 4 peroxidases) in a 3^rd^ category, which were considered to be involved in reactive oxygen species (ROS) detoxification. Another 6 contigs in the 4^th^ category represented four types of transcription factors. The remaining 70 contigs with unknown functions or with functions involved in other processes were grouped into the 5^th^ category and listed in Table S2. We focused on the up-regulated contigs in later studies although the down-regulated contigs shown in Table S2 might also be important.

**Table 1:** Expression levels of Cd up-regulated contigs in HE shoots.

Contig Name	Cont	Cd	FC	q-value	Brief Description
Phenylpropanoids Biosynthesis, Cell Wall Deposition and Modification					
Sa_Contig04589	0.4	23.0	65.3	2.4E-08	Isoflavone 2′-hydroxylase
Sa_Contig14694	1.1	40.5	38.3	3.8E-08	Laccase1–1
Sa_Contig01582	1.0	35.3	34.6	2.6E-09	Laccase 1–2
Sa_Contig00501	5.1	98.7	19.3	7.5E-10	Tyrosine aminotransferase
Sa_Contig15625	0.7	13.1	18.6	9.5E-06	Phenylalanine ammonia-lyase
Sa_Contig21041	7.3	92.4	12.7	3.0E-08	Tyrosine aminotransferase
Sa_Contig15461	3.1	32.1	10.3	2.0E-05	Endo-1,3-beta-glucanase
Sa_Contig10935	10.4	93.7	9.0	1.2E-04	Tyrosine aminotransferase
Sa_Contig13861	3.9	25.8	6.7	1.2E-05	Cinnamate 4-hydroxylase
Sa_Contig15722	4.4	25.4	5.8	8.4E-05	4-coumarate–CoA ligase 1
Sa_Contig26445	4.8	24.6	5.2	3.2E-03	Flavonol 4-reductase
Sa_Contig12913	11.1	43.7	3.9	2.7E-03	Cinnamyl alcohol dehydrogenase
Sa_Contig08243	27.2	69.0	2.5	1.1E-03	ABC transporter B family member 19
Sa_Contig00509	15.5	36.4	2.3	7.0E-06	ABC transporter B family member 19
Sa_Contig10427	45.4	99.3	2.2	1.6E-04	Cinnamate 4-hydroxylase
Sa_Contig10508	354.9	773.2	2.2	3.7E-24	Xyloglucan endotransglucosylase
Sa_Contig12362	104.6	224.2	2.1	2.8E-05	Cellulose synthase-like protein D2
Sa_Contig09979	296.8	604.9	2.0	5.4E-09	Xyloglucan endotransglucosylase
Sa_Contig12072	1346.3	2648.3	2.0	3.7E-45	Xyloglucan endotransglucosylase
Sa_Contig10818	188.7	370.3	2.0	4.1E-07	Cellulose synthase A subunit 1
Metal Transport, Metal Ligand Synthesis and Metal-ligand Transport					
Sa_Contig14529	24.0	135.4	5.6	1.8E-05	Metal transporter Nramp4
Sa_Contig03765	20.1	90.8	4.5	2.8E-03	Metal transporter Nramp2
Sa_Contig30461	30.9	135.0	4.4	2.1E-04	Metal transporter Nramp3
Sa_Contig10390	35.2	114.9	3.3	5.5E-06	Metal transporter Nramp3
Sa_Contig06552	2.4	14.1	5.9	2.1E-04	Metal-nicotianamine transporter YSL3
Sa_Contig08963	19.6	76.0	3.9	2.2E-13	Oligopeptide transporter 3
Sa_Contig11841	25.9	77.3	3.0	3.5E-03	Oligopeptide transporter 3
Sa_Contig43609	44.9	111.4	2.5	5.2E-05	S-adenosylmethionine synthetase 2
Sa_Contig07003	24.3	59.8	2.5	9.4E-04	Methylenetetrahydrofolate reductase 2
Sa_Contig09892	40.3	85.1	2.1	2.2E-03	Probable peptide transporter
Reactive Oxygen Species Detoxification					
Sa_Contig48718	0.3	131.7	512.6	7.1E-05	1-Cys peroxiredoxin PER1
Sa_Contig12607	1.0	161.8	167.8	1.4E-12	1-Cys peroxiredoxin PER1
Sa_Contig52040	0.3	27.5	83.7	6.5E-05	Peroxidase 53
Sa_Contig14027	12.0	106.5	8.8	1.4E-11	Cationic peroxidase 2
Sa_Contig17598	7.7	67.9	8.8	3.8E-05	Peroxidase N1
Sa_Contig42088	283.9	694.2	2.4	1.9E-09	Peroxidase 42
Transcriptional Gene Regulation					
Sa_Contig23581	0.6	55.1	91.1	3.0E-04	Transcription factor ORG2
Sa_Contig35894	0.6	44.3	80.4	3.4E-08	Transcription factor ORG2
Sa_Contig12361	5.5	46.9	8.6	1.7E-04	Transcription factor RAP2.3
Sa_Contig04816	18.7	49.2	2.6	4.5E-03	Transcription factor RF2b
Sa_Contig12226	48.9	101.4	2.1	1.9E-03	Transcription factor HSFC-1
Sa_Contig10599	98.4	193.1	2.0	5.2E-10	Transcription factor ERF9

Notes: Expression abundance of each contig is the mean of three biological replicates and shown in RPKM (Reads Per Kilobase of exon model per Million total reads in sample). Fold Change (FC) = [Cd ]/[Cont]. The rest of 70 contigs in the 5^th^ category is included in Table S2.

To evaluate the validity of Illumina/Solexa data, 15 Cd-induced genes were randomly selected from Table 1 and their expression levels in Cont and Cd were examined by quantitative RT-PCR (qRT-PCR). As expected, the expression pattern of those contigs obtained from qRT-PCR was very highly correlated to the Illumina/Solexa sequencing results with a correlation coefficient of 0.9235 (Figure S5).

### Comparison of Gene Expression in Shoots of Two Contrasting *S. alfredii* Hance Ecotypes

**Figure 5:**
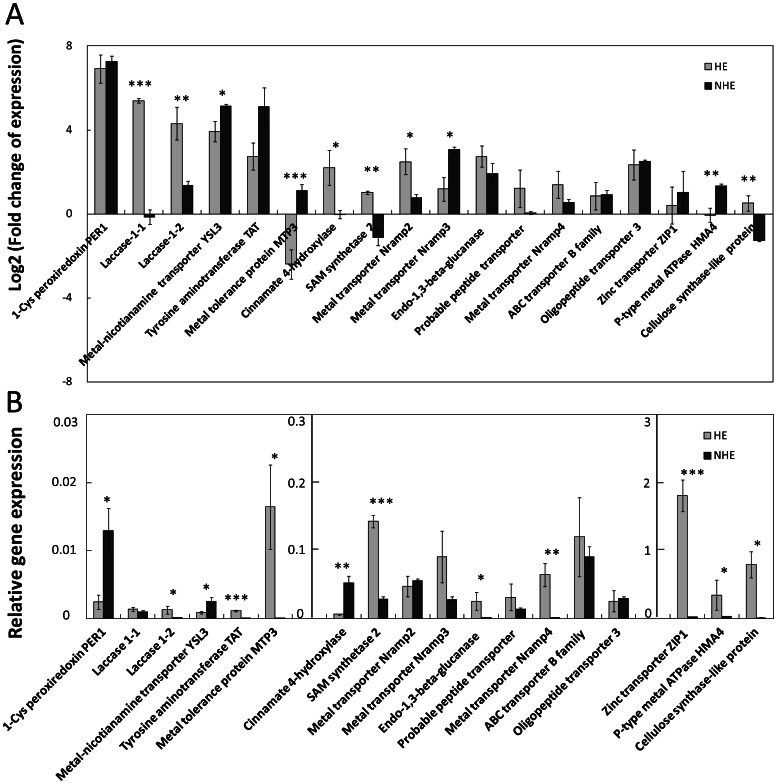
Comparison of gene expression in *S. alfredii* Hance hyper-accumulation ecotype (HE) and non-hyperaccumulation ecotype (NHE). (A) Transcriptional responses of two ecotypes to Cd exposure. Fold change of expression is the relative gene expression level in Cd treatment divided by the value in the control. (B) Relative gene expression levels of two ecotypes under normal growth condition. The relative gene expression is the expression level normalized to a constitutively expressed gene *Actin*. P-type metal ATPase HMA4: *Sa_Contig11685*; Zinc transporter ZIP1: *Sa_Contig10290*; Metal tolerance protein MTP3: *Sa_Contig47062*. The contig numbers for other genes are listed in Table 1. P values are indicated as follows: *P<0.05; **P<0.01; ***P<0.001. Bars depict SE (n = 3).

Previously, the physiological properties of the non-hyperaccumulating ecotype (NHE) was compared with the hyperaccumulating ecotype (HE) when grown on media supplemented with different concentrations of Cd [33]. In the current study, the HE and NHE plants were cultivated hydroponically and supplied with subtoxic Cd concentration for the same period [8], [30]. No morphological difference was observed after Cd exposure. qRT-PCR was employed to compare the gene expression levels between HE and NHE plants in response to prolonged Cd exposure. Several genes were selected based on the current RNA-seq results and three previously reported metal-tolerance-associated genes were also included (Figure 5A). Among them, four contigs (*Sa_Contig14694*, laccase 1–1; *Sa_Contig01582*, laccase 1–2; *Sa_Contig13861*, cinnamate 4-hydroxylase; *Sa_Contig03765*, metal transporter NRAMP2) were more induced in HE than that in NHE (P<0.05); two contigs (*Sa_Contig43609*, SAM synthetase; *Sa_Contig12362*, cellulose synthase) were up-regulated in HE while down-regulated in NHE. In contrast, three contigs (*Sa_Contig06552*, metal-nicotianamine transporter YSL3; *Sa_Contig30461*, metal transporter NRAMP3; *Sa_Contig11685*, P-type metal ATPase HMA4) were more induced in NHE than that in HE (P<0.05). Eight other contigs (*Sa_Contig12607,* 1-Cis peroxiredoxin; *Sa_Contig00501*, tyrosine aminotransferase TAT; *Sa_Contig15461*, endo-1,3-beta-glucanase; *Sa_Contig09892*, probable peptide transporter; *Sa_Contig14529*, metal transporter NRAMP4; *Sa_Contig08243*, ABC transporter B family member 19; *Sa_Contig08963*, Oligopeptide transporter 3; *Sa_Contig10290*, zinc transporter ZIP1) were up-regulated by Cd with no significant difference between two ecotypes (P<0.05). Interestingly, one contig (*Sa_Contig47062*, metal tolerance protein MTP3) was down-regulated in HE while up-regulated in NHE. In order to know whether Cd hyper-tolerance in HE is correlated to constitutive higher expression of certain genes, the expression levels of 18 aforementioned contigs were compared between HE and NHE under normal growth condition (Figure 5B). Except 1-Cis peroxiredoxin (*Sa_Contig12607*), metal-nicotianamine transporter YSL3 (*Sa_Contig06552*) and cinnamate 4-hydroxylase (*Sa_Contig13861*), of which expression levels were higher in NHE than that in HE (P<0.05), nine other contigs (*Sa_Contig01582*, laccase 1–2; *Sa_Contig00501*, tyrosine aminotransferase TAT; *Sa_Contig47062*, metal tolerance protein MTP3; *Sa_Contig43609*, SAM synthetase; *Sa_Contig15461*, endo-1,3-beta-glucanase; *Sa_Contig14529*, metal transporter NRAMP4; *Sa_Contig10290*, zinc transporter ZIP1; *Sa_Contig11685*, P-type metal ATPase HMA4; *Sa_Contig12362*, cellulose synthase) related to metal transport and cell wall modification process were constitutively expressed at higher levels in HE than that in NHE (P<0.05). The rest of the six contigs (*Sa_Contig14694*, laccase 1–1; *Sa_Contig03765*, metal transporter NRAMP2; *Sa_Contig30461*, metal transporter NRAMP3; *Sa_Contig09892*, probable peptide transporter; *Sa_Contig08243*, ABC transporter B family member 19; *Sa_Contig08963*, Oligopeptide transporter 3) expressed at similar level in both ecotypes under normal growth condition (P<0.05).

## Discussion

Heavy metal hyperacculuators have extreme lifestyles in which high levels of toxic elements are accumulated in these plants without obvious toxicity symptoms. The *Crassulaceae* family plant *S. alfredii* Hance (HE) is the only identified Zn and Cd hyperaccumulating plant to date that does not belong to *Brassicaceae* family [6].The major strategy hyperaccumulators have used is to protect themselves by compartmentalization of metal ions in the cell. Several contigs related to cell wall deposition and modifications were identified as Cd responsive genes in *S. alfredii* Hance (HE) shoots in the current study. Lignin is a complex phenylpropanoid polymer deposited in the secondary cell walls [31]. The formation of phenylpropanoids starts from phenylalanine and involves three important enzymes: phenylalanine ammonia-lyase, cinnamate 4-hydroxylase, and 4-coumaroyl CoA-Ligase. Cinnamyol alcohol dehydrogenase is considered as the key enzyme in linking the central metabolite 4-coumaroyl CoA to lignin production. Tyrosine aminotransferase catalyzes the conversion of tyrosine to 4-hydroxyphenylpyruvic acid, which is an intermediate in the metabolism of phenylalanine [32]. The ATP-binding cassette (ABC) subfamily B transporters (ABCB) and multi-copper-containing glycoprotein laccases have also been demonstrated to be associated with cell wall lignifications [33], [34]. Contigs encoding the above-mentioned six enzymes and one ABCB gene were up-regulated by Cd in HE shoots (Table 1). Lignin accumulates between cellulose, hemicellulose and pectin components in the cell wall. Cellulose synthase (CESA) forms large membrane complexs and is responsible for the cellulose formation. Xyloglucan is the major hemicellulose in cell walls and crosslinks microfibrils. Xyloglucan endotransglucosylase (XTH) has the ability to breakdown xyloglucan strands that are not tightly linked to cellulose and to integrate new xyloglucans into the cell walls. Beside XTH, endo-1,3(4)-beta-glucanases in glycoside hydrolase family also have the potential to function in cell wall loosening [35]. Contigs encoding CESA, XTH and endo-1,3-beta-glucanase were also up-regulated by Cd in HE shoots (Table 1). Furthermore, genes encoding laccase, cinnamate 4-hydroxylase, and cellulose synthase were not only more induced but also expressed at higher basal level in HE than that in NHE (Figure 5). Although genes encoding endo-1,3(4)-beta-glucanase and tyrosine aminotransferase were both up-regulated by Cd with similar fold change in HE and NHE, they were constitutively expressed at higher levels in HE than that in NHE (Figure 5). These results indicate that Cd tolerance in HE shoots is probably associated with cell wall formation/modification. In a previous physiological study, we found that the highest amount of Cd was associated with cell wall fraction in HE shoots [36].

Vacuolar sequestration in leaves is a common mechanism for heavy metal compartmentalization in the hyperacculators *A. halleri* and *T. caerulescens* [6], [11]. Cation-diffusion facilitator genes (also known as MTPs) or P-type ATPase genes (e.g. AtHMA3) are important for vacuolar sequestration [6], however none were found to be up-regulated by Cd in HE shoots in the current study. On the contrary, metal tolerance protein MTP3 was down-regulated in HE but up-regulated in NHE. Two NRAMP (nature resistance associated with microphage) family genes (NRAMP2 and NRAMP3) functioning in vacuolar efflux of heavy metals [6] were significantly up-regulated by Cd in both HE and NHE. Although basal expression levels of NRAMP2 and NRAMP3 were not significantly different (P<0.05) between HE and NHE, NRAMP2 was more highly induced in HE while NRAMP3 was more induced in NHE. Another NRAMP gene NRAMP4 was also identified in our experiments, of which basal expression level was much higher in HE than that in NHE. Nevertheless, NRAMP3 expression level in HE was similar to the one in NHE while NRAMP2 and NRAMP4 expression levels were much higher in HE than that in NHE under Cd treated condition in our experiments. The P-type metal ATPase, HMA4, is involved in cytosolic metal efflux and xylem loading/unloading [9]. In the current study, one heavy metal transporter HMA4 was more up-regulated by Cd in NHE than that in HE. However, HMA4 was more expressed in HE than that in NHE (10.2 fold higher in Cont and 2.7 fold higher in Cd, respectively). We also examined the expression of one ZIP family gene metal transporter ZIP1. There was no significant difference in terms of up-regulation ratio between HE and NHE, but ZIP1 expression level was again much higher in HE than that in NHE (98.8 fold higher in Cont and 97.7 fold higher in Cd, respectively). Root-to-shoot translocation of Cd in *S. alfredii* Hance (HE) was found to be enhanced in our previous physiological study [16]. It would be interesting to know in the future whether the high expression level of HMA4 and ZIP1 in HE could contribute to such active translocation process.

In summary, we have generated a large collection of annotated transcript contigs from the multi-heavy-metal accumulator *S. alfredii* Hance (HE) shoots, which should provide more opportunities for studying heavy metal hyperaccumlation. Our shoot transcriptome analysis has revealed that several genes related to cell wall modification, metal translocation and remobilization are highly induced in *S. alfredii* Hance (HE). Subsequent qRT-PCR results have demonstrated that there are significant differences in the gene expression pattern between HE and NHE shoots under both normal growth condition and Cd-treated conditions. The sequencing data presented here will also aid in elucidating the functional roles of the constitutively expressed genes in Cd hyper-tolerance in *S. alfredii* Hance (HE).

## Materials and Methods

### Plant Growth and Treatment

The hyperaccumulating ecotype *S. alfredii* Hance (HE) was obtained from an old Pb/Zn mine area in Zhejiang Province in China and the non-hyperaccumulating ecotype *S. alfredii* Hance (NHE) was obtained from a tea plantation of Hangzhou in Zhejiang Province. The plants described here did not involve endangered or protected species. No specific permissions were required for collection of samples in these locations. Plants were vegetatively propagated to ensure homogeneity and cultured hydroponically in basal nutrient solution [14] supplied with or without 100 µM CdCl_2_ for HE and 5 µM CdCl_2_ for NHE for 8 days. Samples (three plants per sample) were separated into shoots and roots for RNA sequencing or qRT-PCR or elemental analysis. There were three biological replicates in each experiment. Cd concentrations were measured routinely by Inductively Coupled Plasma Mass Spectroscopy (ICP-MS) (Agilent, USA). All the data was statistically analyzed using student’s t-test or ANOVA.

### Sequence Generation, Mapping and Assembly

For deep sequencing, total RNA was extracted from HE shoots with a RNAout kit (TANDZ, China) and quality checked with an Agilent 2100 Bioanalyzer (*Agilent*, USA). mRNA was purified with Micropoly(A)PuristTM mRNA purification kit (Ambion, USA) following the DNase I digestion (Ambion, USA). First-strand cDNA was synthesized with superscript III reverse transcriptase (Invitrogen, USA) and GsuI-oligo dT. mRNA was biotinylated after being oxidized at its 5′ end with NaIO4 (Sigma, USA) and removed with Dynal M280 magnetic streptavidin beads (Invitrogen, USA) after alkaline digestion. Adaptors were added to the 5′-end of first-strand cDNA with DNA ligase (TaKaRa, Japan) and second-strand cDNA was synthesized with Ex Taq polymerase (TaKaRa, Japan). polyA tails and 5′ end adaptors were removed with GsuI digestion. cDNA was purified with Ampure beads (Agencourt, USA) after sonication (Fisher, USA). 454 and Illumina/Solexa libraries were constructed with GS DNA Library Preparation kit (Roche Applied Science, USA) and TruSeqTM DNA Sample Prep Kit – Set A (Illumine, USA), respectively. Deep sequencing was performed with Roche’s 454 Genome Sequencer FLX and Illumina/Solexa Genome Analyzer II platforms in the Chinese National Human Genome Center at Shanghai. Sequences from 454 sequencing were assembled with the Newbler Assembler [37]. Illumina/Solexa reads were mapped to the 454-derived contigs with bowtie [29], and the remaining unmapped reads were separately assembled using Velvet/Oases [22]. All the contigs were finally assembled in CAP3 [23]. Default settings were used in the above-mentioned programs. To minimize the alternative splicing effects on assembly, the longest contig/isotig was selected for each isogroup, or the best match was selected when potential paralogs were presented. Microsatellites (SSRs) were obtained in SciRoKo (v3.4) with misa (perfect) and mmvp (mismatch allowed) modes [38]. Enrichment of KEGG pathways for a given gene list was calculated using a classical hypergeometric distribution statistical comparison of a query gene list against a reference gene list. For homologous protein comparison, sequence similarity between *S. alfredii* Hance (HE) contigs and other sequenced plants were obtained by BlastP using different E-value cutoffs [39].

### Gene Expression Analysis, Quantitative RT-PCR and Phylogenic Analysis

Low quality reads were detected and removed with the FASTQ Quality Trimmer (−t 5, −l 50) in FASTX-Toolkit after reads containing undetermined bases (N) were discarded, the clean Illumina/Solexa reads in each sample were mapped to the assembled contigs and normalized to RPKM [29]. When reads were mapped to multiple contigs, only one match was randomly selected. Gene expression levels was determined with the MARS (MA-plot-based method with Random Sampling) model with DEGseq package [40]. The FDR was controlled using q-value (q = 0.005) to identify significant differences between treatments [40]. For qRT-PCR analysis, total RNA from three biological replicates was isolated (Qiagen, Germany) from shoots of HE and NHE, and reverse transcribed using the Supertranscript III RT kit (Invitrogen, USA) according to the manufacturer’s instructions. For primer design (Table S4), blast searches were conducted against the Arabidopsis nucleotide database with *S. alfredii* Hance (HE) contig sequences, and primers were selected from sequences in the sequence-conserved regions. Melting curves were monitored to ensure good amplification in both ecotypes. qRT-PCR was performed with the Mastercycler**®** ep realplex2 instrument (Eppendorf, Germany) with SsoFast^TM^EvaGreen**®** Supermix kit (Bio-Rad, USA). Expression levels were calculated relative to actin using a comparative threshold cycle method (CT method) [41]. C3-, C4- and CAM-type PEPC protein sequences were aligned together with the *S. alfredii* PEPC contigs in ClustalX and maximum parsimony tree was constructed in MEGA (version 5.05) with standard settings. The accession number of plant PEPCs is listed in Table S5.

### Data Deposition

The RNA-seq results reported in this paper have been deposited into EMBL-Bank under the accession number: E-MTAB-1011; ArrayExpress under the accession number: E-MTAB-934. The assembled contigs were assigned with the accession number from HE717106 to HE774267.

## Supporting Information

Figure S1 **Sequence size distribution.** (A) Length distribution of sequences from Roche’s 454 sequencing. (B) Length distribution of assembled contigs from Illumina/Solexa sequencing.
(PDF)

Figure S2 **Pathway analysis of** ***S. alfredii*** **Hance (HE) contigs.**
(PDF)

Figure S3 **Phylogenetic analysis of PEPC genes.** Phylogenetic hypothesis was derived from maximum parsimony analysis of the C-terminal fragments of PEPCs from *S*. *alfredii* (Sa_) and *Arabidopsis thaliana* (At_), *Zea mays* (Zm_), *Sorghum vulgare* (Sv_), *Saccharum officinarum* (So_), *Mesembryanthemum crystallinum* (Mc_), *Kalanchoë blossfeldiana* (Kb_), *Clusia venosa* (Cv_), *Clusia rosea* (Cr_), *Clusia schomburgkiana* (Cs_), *Clusia hilariana* (Ch_) and *Clusia aripoensis* (Ca_). The tree was rooted with *Saccharum officinarum*. Accession numbers are listed in Table S5.
(PDF)

Figure S4 **Partial protein sequence alignment of CAM-type PEPCs.** Accession numbers are listed in Table S5. Identical amino acids are marked with stars (*) under the lines, colons and dots indicate different amino acids with strong and weak similarity, respectively.
(PDF)

Figure S5 **Validation of RNA-seq results by qRT-PCR.** The gene expression level of each contig in the hyperaccumulation ecotype (HE) was quantified and normalized to the *actin* control. The fold change of expression is the gene expression level in Cd treatment normalized to that in CK. Annotation of each contig is listed in Table 1. Bars depict SE (n = 3).
(PDF)

Table S1 **Overview of the Illumina/Solexa sequencing.**
(DOC)

Table S2 **Contigs differentially regulated by Cd treatment.**
(DOC)

Table S3 **List of PEPCs in** ***S. alfredii*** **Hance.**
(DOC)

Table S4 **List of primers used for qRT-PCR.**
(DOC)

Table S5 **Accession numbers of plant PEPCs for sequence alignment.**
(DOC)

Dataset S1 **Simple sequence repeats found in the study.**
(XLS)
