# Supplementary material for: Transcriptomic Analysis of Cadmium Stress Response in the Heavy Metal Hyperaccumulator Sedum alfredii Hance
Source: PLoS One. 2013 Jun 3;8(6):e64643. doi: 10.1371/journal.pone.0064643 (PMC3670878; doi:10.1371/journal.pone.0064643)
Supplement: Figure S1 — Sequence size distribution. (A) Length distribution of sequences from Roche’s 454 sequencing. (B) Length distribution of assembled contigs from Illumina/Solexa sequencing. (PDF) [file pone.0064643.s001.pdf]

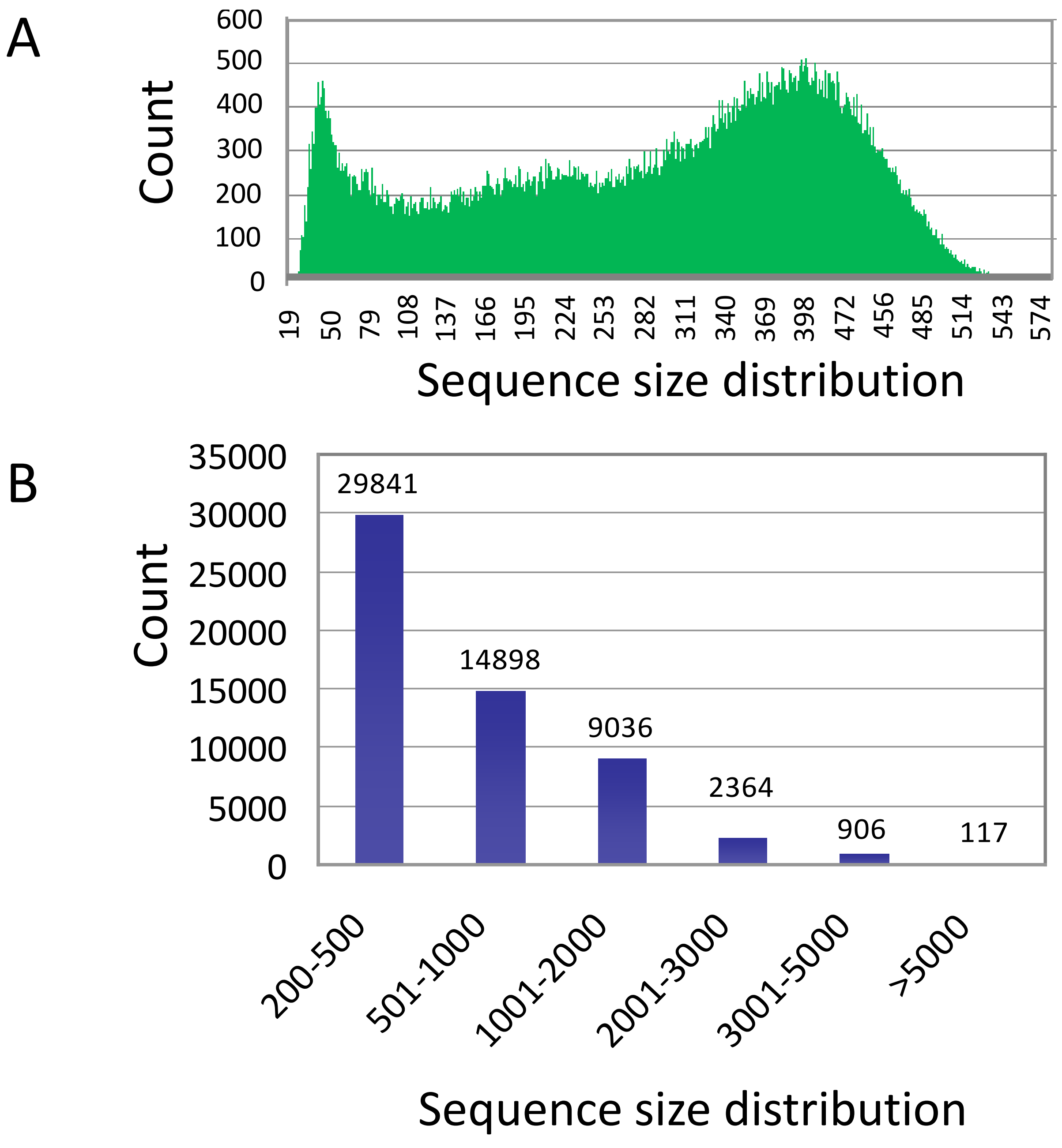

Figure S1 Sequence size distribution. (A) Length distribution of sequences from Roche's 454 sequencing. (B) Length distribution of assembled contigs from Illumina/Solexa sequencing.
