# Supplementary figures and images for: Transcriptomic Analysis of Cadmium Stress Response in the Heavy Metal Hyperaccumulator Sedum alfredii Hance
Source: PLoS One. 2013 Jun 3;8(6):e64643. doi: 10.1371/journal.pone.0064643 (PMC3670878; doi:10.1371/journal.pone.0064643)

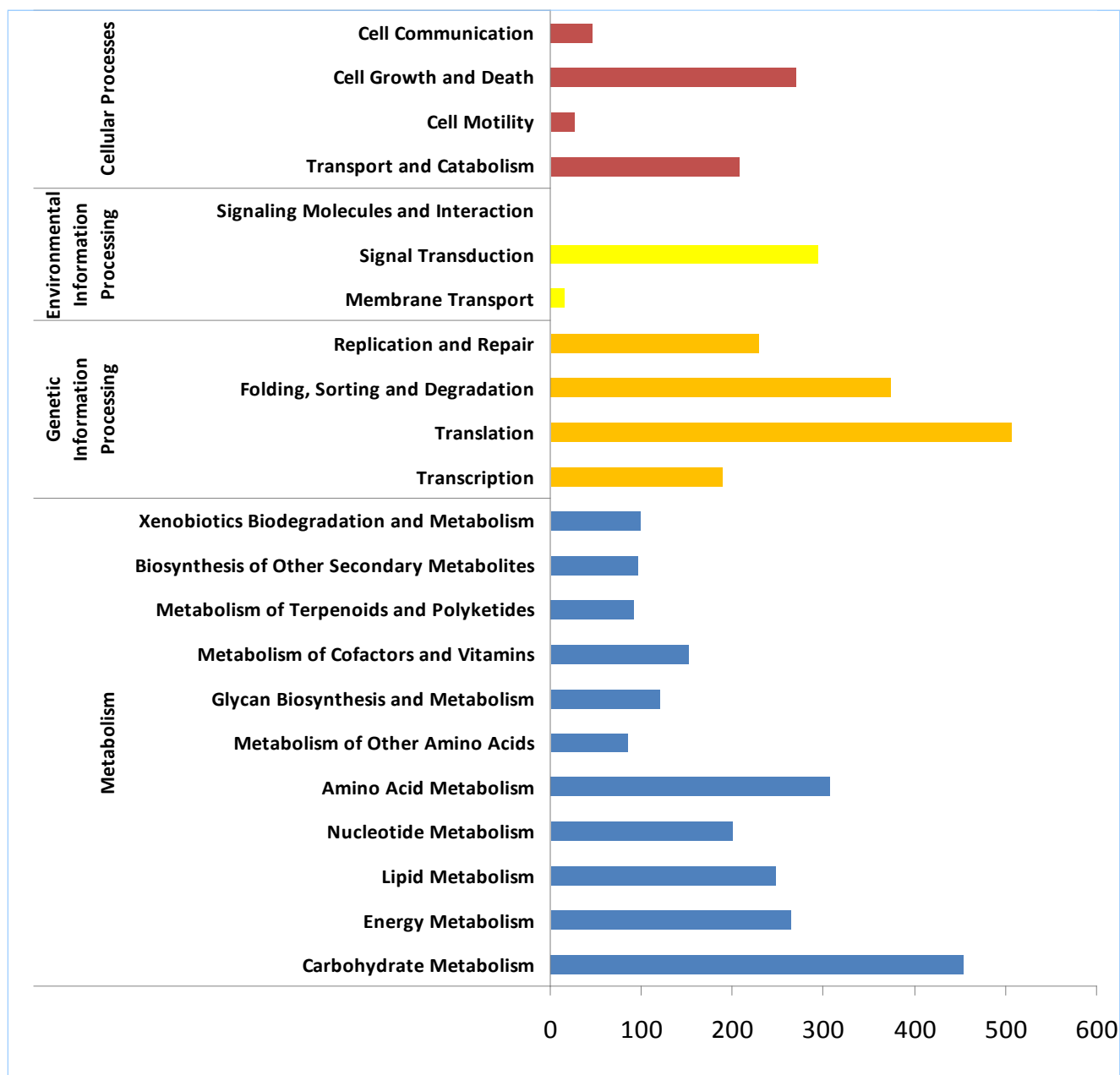

**Figure S2 Pathway analysis of *S. alfredii* Hance (HE) contigs.**

Supplement: Figure S2 — Pathway analysis of S. alfredii Hance (HE) contigs. (PDF) [file pone.0064643.s002.pdf]
