# Supplementary material for: Transcriptomic Analysis of Cadmium Stress Response in the Heavy Metal Hyperaccumulator Sedum alfredii Hance
Source: PLoS One. 2013 Jun 3;8(6):e64643. doi: 10.1371/journal.pone.0064643 (PMC3670878; doi:10.1371/journal.pone.0064643)
Supplement: Figure S3 — Phylogenetic analysis of PEPC genes. Phylogenetic hypothesis was derived from maximum parsimony analysis of the C-terminal fragments of PEPCs from S. alfredii (Sa_) and Arabidopsis thaliana (At_), Zea mays (Zm_), Sorghum vulgare (Sv_), Saccharum officinarum (So_), Mesembryanthemum crystallinum (Mc_), Kalanchoë blossfeldiana (Kb_), Clusia venosa (Cv_), Clusia rosea (Cr_), Clusia schomburgkiana (Cs_), Clusia hilariana (Ch_) and Clusia aripoensis (Ca_). The tree was rooted with Saccharum officinarum. Accession numbers are listed in Table S5. (PDF) [file pone.0064643.s003.pdf]

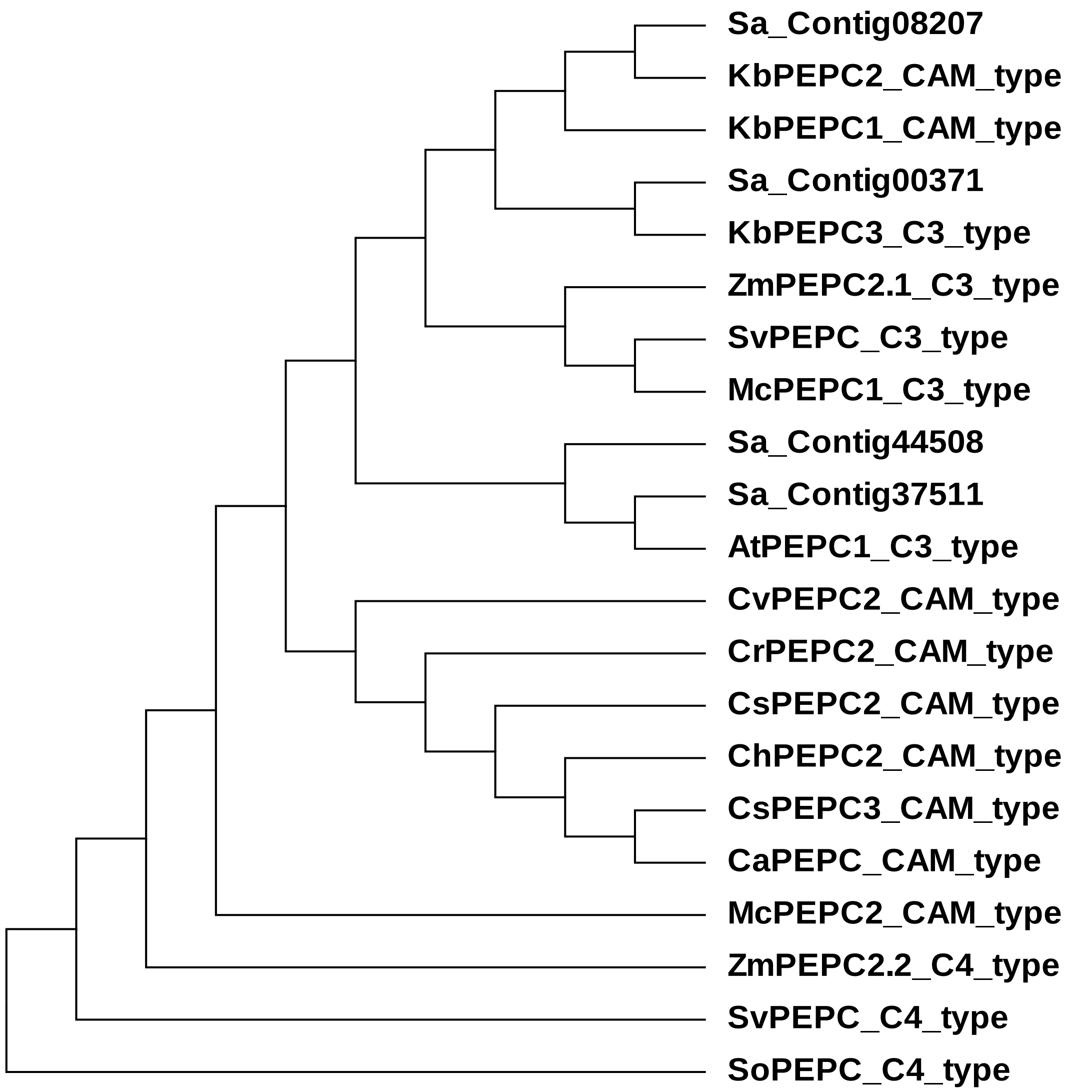

Figure S3 Phylogenetic analysis of PEPC genes. Phylogenetic hypothesis was derived from maximum parsimony analysis of the C-terminal fragments of PEPCs from *S. alfredii* (Sa_) and *Arabidopsis thaliana* (At_), *Zea mays* (Zm_), *Sorghum vulgare* (Sv_), *Saccharum officinarum* (So_), *Mesembryanthemum crystallinum* (Mc_), *Kalanchoë blossfeldiana* (Kb_), *Clusia venosa* (Cv_), *Clusia rosea* (Cr_), *Clusia schomburgkiana* (Cs_), *Clusia hilariana* (Ch_) and *Clusia aripoensis* (Ca_). The tree was rooted with *Saccharum officinarum*. Accession numbers are listed in Table S5.
