# Supplementary material for: Transcriptomic Analysis of Cadmium Stress Response in the Heavy Metal Hyperaccumulator Sedum alfredii Hance
Source: PLoS One. 2013 Jun 3;8(6):e64643. doi: 10.1371/journal.pone.0064643 (PMC3670878; doi:10.1371/journal.pone.0064643)
Supplement: Figure S4 — Partial protein sequence alignment of CAM-type PEPCs. Accession numbers are listed in Table S5. Identical amino acids are marked with stars (*) under the lines, colons and dots indicate different amino acids with strong and weak similarity, respectively. (PDF) [file pone.0064643.s004.pdf]

```
CvPEPC2_CAM_type    FHGRGGTVGRGGGPSHLAILSQPPDTIHGLLRVTVQGEVIEQSFGEEHLCFRTLQRFTAA 60
CsPEPC2_CAM_type    FHGRGGTVGRGGGPSHLAILSQPPDTIHGLLRVTVQGEVIEQSFGEEHLCFRTLQRFTAA 60
CrPEPC2_CAM_type    FHGRGGTVGRGGGPSHLAILSQPPDTIHGSLRVTVQGEVIEQSFGEEHLCFRTLQRFTAA 60
ChPEPC2_CAM_type    FHGRGGTVGRGGGPSHLAILSQPPDTIRGSLRVTVQGEVIEQSFGEEHLCFRTLQRFTAA 60
CsPEPC3_CAM_type    FHGRGGTVGRGGGPSHLAILSQPPDTIHGSLRVTVQGEVIEQSFGEEHLCFRTLQRFTAA 60
CaPEPC_CAM_type     FHGRGGTVGRGGGPSHLAILSQPPDTIHGSLRVTVQGEVIEQSFGEEHLCFRTLQRFTAA 60
McPEPC1_CAM_type    FHGRGGTVGRGGGPTHLAILAQPAETIGGSLRVTIQGEVIEQSFGEQHLCFRTLQRYTAA 60
KbPEPC1_CAM_type    FHGRGGTVGRGGGPTHLAILSQPADTIQGSLRVTIQGEVIERSFGEAQLCFKTLQRYTAA 60
KbPEPC2_CAM_type    FHGRGGTVGRGGGPTHLAILSQPADTIQGSLRVTIQGEVIERSFGEAQLCFKTLQRYTAA 60
Sa_Contig08207      FHGRGGTVGRGGGPTHLAILSQPPDTVQGSLRVTVQGEVIERSFGESQLCFRTLQRFTAA 60
                    **************:*****:**.:*: * ****:******:**** :***:****:***

CvPEPC2_CAM_type    TLEHGMHPPVSPKPEWRA-LLDEMAVIATEEYRSIVFKEPQFVEYFRLATPELEYGRMNI 119
CsPEPC2_CAM_type    TLEHGMHPPVSPKPEWRA-LLDEMAVIATEEYRSIVFKEPQFVEYFRLATPELEYGRMNI 119
CrPEPC2_CAM_type    TLEHGMHPPVSPKPEWRA-LLDEMAVIATEEYRSIVFKEPKFVEYFRLATPELEYGRMNI 119
ChPEPC2_CAM_type    TLEHGMHPPVSPKPEWRA-LLDEMAVIATEEYRSIVFKEPQFVEYFRLATPELEYGRMNI 119
CsPEPC3_CAM_type    TLEHGMHPPVSPKPEWRA-LLDEMAVVATEEYRSIVFKEPQFVEYFRLATPELEYGRMNI 119
CaPEPC_CAM_type     TLEHGMHPPVSPKPEWRA-LLDEMAVVATEEYRSIVFKEPQFVEYFRLATPELEYGRMNI 119
McPEPC1_CAM_type    TLEHGMNPPKSPKPEWRA-LLDQMAVVATEEYRSIVFKEPRFVEYFRLATPETEYGRMNI 119
KbPEPC1_CAM_type    TLEHGMIPPSSPKQECRACLMDEMAVVTTERYRSIVFREPRFVEYFRLATPELEYGRMNI 120
KbPEPC2_CAM_type    TLEHGMIPPSSPKQEWRACLMDEMAVVTTERYRSIVFREPRFVEYFRLATPELEYGRMNI 120
Sa_Contig08207      TLEHGMNPPLAPKQEWRD-LMDEMAVVATEHYRSIVFEEPRFVEYFRLATPEMEYGRMNI 119
                    ****** ** :** * *  *:*:***::**.******.**:*********** *******

CvPEPC2_CAM_type    GSRPSKRKPSGGIESLRAIPWIFAWTQTRFHLPVWLGFGAAFKHIIKKDIRNLYVLQEMY 179
CsPEPC2_CAM_type    GSRPSKRKPSGGIESLRAIPWIFAWTQTRFHLPVWLGFGAAFKHIIKKDIRNLHVLQEMY 179
CrPEPC2_CAM_type    GSRPSKRKPSGGIESLRAIPWIFAWTQTRFHLPVWLGFGAAFKHIIKKDIRNLHVLQEMY 179
ChPEPC2_CAM_type    GSRPSKRKPSGGIESLRAIPWIFAWTQTRFHLPVWLGFGAAFKHIIKKDIRNLHVLQEMH 179
CsPEPC3_CAM_type    GSRPSKRKPSGGIESLRAIPWIFAWTQTRFHLPVWLGFGAAFKHIIKKDIRNLHVLQEMY 179
CaPEPC_CAM_type     GSRPSKRKPSGGIESLRAIPWIFAWTQTRFHLPVWLGFGAAFKHIIKKDIRNLHVLQEMY 179
McPEPC1_CAM_type    GSRPSKRKPSGGIESLRAIPWIFAWTQTRFHLPVWLGVGGALKHVLEKDIRNHNMLRDMY 179
KbPEPC1_CAM_type    GSRPSKRKPSGGIESLRAIPWIFAWTQTRFHLPVWLGFGEAFRHVIDKDNKNLLMLQQMY 180
KbPEPC2_CAM_type    GSRPSKRKPSGGIESLRAIPWIFAWTQTRFHLPVWLGFGEAFRHVIDKDNKNLLMLQQMY 180
Sa_Contig08207      GSRPSKRKPSGGIESLRAIPWIFAWTQTRFHLPVWLGFGEAFRHIIDKDNKNLLMLQQMY 179
                    *************************************.* *::*::.** :*  :*::*:

CvPEPC2_CAM_type    NAWPFFRVTIDMLEMVFAKGNPGIAALYDKLLVSEDLWTFGEKLRTNYEETKKLLLQIAG 239
CsPEPC2_CAM_type    NAWPFFRVTIDMLEMVFAKGDPGIAALYDKLLVSEDLWTFGEELRTNYEETKKLLLQIAG 239
CrPEPC2_CAM_type    NAWPFFRVTIDMLEMVFAKGDPGIAALYDKLLVSEELWTFGEKLRTNYEETKKLLLQIAG 239
ChPEPC2_CAM_type    NAWPFFRVTIDLLEMVFAKGDPGIAALYDKLLVSEDLWTFGEKLRTNYEETKTFLLQIAG 239
CsPEPC3_CAM_type    NAWPFFRVTIDMLEMVFAKGNPEIAALYDKLLVSEDLWTFGEKLRTNYEETKKLLLQIAG 239
CaPEPC_CAM_type     NAWPFFRVTIDMLEMVFAKGNPEIAALYDKLLVSEDLWTFGEKLRTNYEETKRLLLQIAG 239
McPEPC1_CAM_type    NNFPFFRVTIDLLEMVFAKGDPEIAALYDKLLVSEELQSFGERLRANYEDTKRRLLEVAG 239
KbPEPC1_CAM_type    NEWPFFRVTIDLVEMVFAKGDPGIAALYDKLLS-EELWPLGEQLRTAYNDTKSYLLKITG 239
KbPEPC2_CAM_type    NEWPFFRATIDLVEMVFAKGDPGIAALYDKLLS-EELWPLGEQLRTAYNDTKSYLLKITG 239
Sa_Contig08207      NEWPFFRVTIDLVEMVFAKGNPGIAALYDKLLVSEDLLALGERLRTAYNDTQGFLLKITG 239
                    * :****.***::*******:* *********  *:* .:**.**: *::*:  **:::*
```

Figure S4 Partial protein sequence alignment of CAM-type PEPCs. Accession numbers are listed in Table S5. Identical amino acids are marked with stars (*) under the lines, colons and dots indicate different amino acids with strong and weak similarity, respectively.
