# Supplementary material for: Transcriptomic Analysis of Cadmium Stress Response in the Heavy Metal Hyperaccumulator Sedum alfredii Hance
Source: PLoS One. 2013 Jun 3;8(6):e64643. doi: 10.1371/journal.pone.0064643 (PMC3670878; doi:10.1371/journal.pone.0064643)
Supplement: Figure S5 — Validation of RNA-seq results by qRT-PCR. The gene expression level of each contig in the hyperaccumulation ecotype (HE) was quantified and normalized to the actin control. The fold change of expression is the gene expression level in Cd treatment normalized to that in CK. Annotation of each contig is listed in Table 1. Bars depict SE (n = 3). (PDF) [file pone.0064643.s005.pdf]

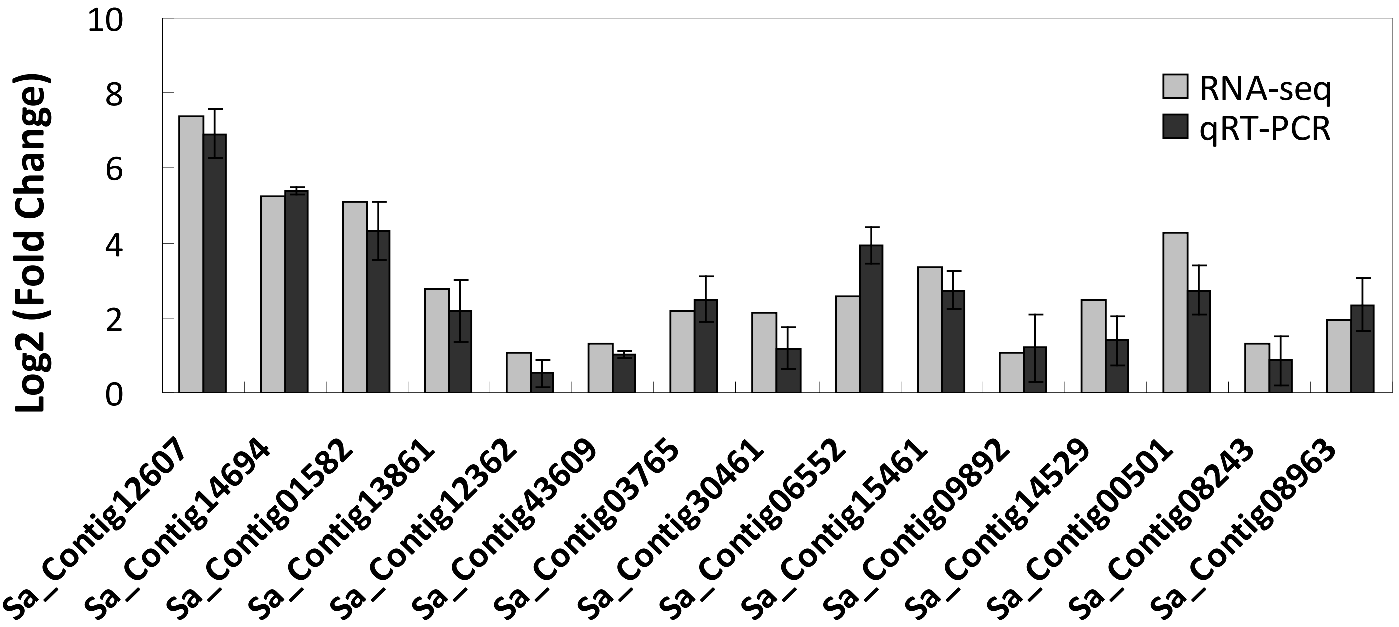

Figure S5 Validation of RNA-seq results by qRT-PCR. The gene expression level of each contig in the hyperaccumulation ecotype (HE) was quantified and normalized to the *actin* control. The fold change of expression is the gene expression level in Cd treatment normalized to that in CK. Annotation of each contig is listed in Table 1. Bars depict SE (n=3)
