# Supplementary material for: Transcriptomic Analysis of Cadmium Stress Response in the Heavy Metal Hyperaccumulator Sedum alfredii Hance
Source: PLoS One. 2013 Jun 3;8(6):e64643. doi: 10.1371/journal.pone.0064643 (PMC3670878; doi:10.1371/journal.pone.0064643)
Supplement: Table S1 — Overview of the Illumina/Solexa sequencing. (DOC) [file pone.0064643.s006.doc]

**Table S1 Overview of the Illumina/Solexa sequencing**

| **sample** | **Cont1** | **Cont2** | **Cont3** | **Cd1** | **Cd2** | **Cd3** |
| --- | --- | --- | --- | --- | --- | --- |
| **Reads (pair)** | **4,099,413** | **9,751,583** | **9,822,807** | **6,431,480** | **6,727,157** | **10,672,407** |
| **Average Length** | **105 (base)** | **97 (base)** | **97 (base)** | **101 (base)** | **100 (base)** | **99 (base)** |
| **Total No of bases** | **860,699,544** | **1,891,357,078** | **1,913,784,422** | **1,293,377,440** | **1,348,743,470** | **2,113,559,836** |
