# Supplementary material for: Transcriptomic Analysis of Cadmium Stress Response in the Heavy Metal Hyperaccumulator Sedum alfredii Hance
Source: PLoS One. 2013 Jun 3;8(6):e64643. doi: 10.1371/journal.pone.0064643 (PMC3670878; doi:10.1371/journal.pone.0064643)
Supplement: Table S2 — Contigs differentially regulated by Cd treatment. (DOC) [file pone.0064643.s007.doc]

**Table S2 Contigs differentially regulated by Cd treatment**

| **Contig Name** | **Cont** | | **Cd** | | **FC** | **q-value** | **Brief Description** |
| --- | --- | --- | --- | --- | --- | --- | --- |
|  | **reads** | **RPKM** | **reads** | **RPKM** |  |  |  |
| **Sa_Contig48718** | **0.3** | **0.3** | **139.3** | **131.7** | **512.6** | **7.1E-05** | **1-Cys peroxiredoxin PER1** |
| **Sa_Contig10825** | **0.3** | **0.1** | **89.3** | **25.2** | **348.0** | **2.4E-03** | **Polyphenol oxidase, chloroplastic** |
| **Sa_Contig12607** | **1.7** | **1.0** | **264.0** | **161.8** | **167.8** | **1.4E-12** | **1-Cys peroxiredoxin PER1** |
| **Sa_Contig23581** | **1.3** | **0.6** | **102.3** | **55.1** | **91.1** | **3.0E-04** | **Transcription factor ORG2** |
| **Sa_Contig52040** | **1.7** | **0.3** | **112.7** | **27.5** | **83.7** | **6.5E-05** | **Peroxidase 53** |
| **Sa_Contig35894** | **2.3** | **0.6** | **171.3** | **44.3** | **80.4** | **3.4E-08** | **Transcription factor ORG2** |
| **Sa_Contig04589** | **3.0** | **0.4** | **189.0** | **23.0** | **65.3** | **2.4E-08** | **Isoflavone 2'-hydroxylase** |
| **Sa_Contig33406** | **5.0** | **3.7** | **282.0** | **215.5** | **58.5** | **3.9E-13** | **Hypothetical protein** |
| **Sa_Contig42219** | **1.7** | **0.5** | **82.0** | **27.6** | **50.9** | **1.0E-03** | **Isease resistance response protein 206** |
| **Sa_Contig22069** | **2.0** | **0.2** | **75.3** | **11.2** | **45.8** | **4.1E-03** | **Lysine histidine transporter-like 8** |
| **Sa_Contig05342** | **0.0** | **0.0** | **93.3** | **22.2** | **43.3** | **2.7E-04** | **Aquaporin TIP2-1** |
| **Sa_Contig14694** | **5.3** | **1.1** | **179.3** | **40.5** | **38.3** | **3.8E-08** | **Laccase 1-1** |
| **Sa_Contig01582** | **6.0** | **1.0** | **205.0** | **35.3** | **34.6** | **2.6E-09** | **Laccase 1-2** |
| **Sa_Contig01443** | **9.7** | **5.2** | **317.3** | **170.4** | **32.6** | **3.3E-15** | **Hypothetical protein** |
| **Sa_Contig26275** | **4.0** | **4.4** | **120.7** | **129.2** | **29.1** | **2.1E-05** | **Hypothetical protein** |
| **Sa_Contig12055** | **6.7** | **2.9** | **113.7** | **60.9** | **21.3** | **2.1E-05** | **Hypothetical protein** |
| **Sa_Contig00501** | **14.3** | **5.1** | **237.0** | **98.7** | **19.3** | **7.5E-10** | **Tyrosine aminotransferase** |
| **Sa_Contig15625** | **7.7** | **0.7** | **145.0** | **13.1** | **18.6** | **9.5E-06** | **Phenylalanine ammonia-lyase** |
| **Sa_Contig49185** | **8.7** | **2.8** | **133.0** | **50.2** | **18.2** | **1.1E-05** | **Isoeugenol synthase 1** |
| **Sa_Contig20291** | **14.7** | **10.3** | **213.7** | **164.2** | **16.0** | **6.8E-09** | **Hypothetical protein** |
| **Sa_Contig56117** | **9.7** | **2.8** | **124.0** | **40.6** | **14.7** | **5.3E-05** | **Isoeugenol synthase 1** |
| **Sa_Contig04579** | **7.0** | **3.5** | **124.3** | **50.2** | **14.3** | **1.5E-04** | **Hypothetical protein** |
| **Sa_Contig16400** | **9.7** | **6.4** | **121.0** | **88.8** | **13.8** | **1.4E-04** | **Hypothetical protein** |
| **Sa_Contig17350** | **6.3** | **7.6** | **123.7** | **99.8** | **13.1** | **4.6E-05** | **Hypothetical protein** |
| **Sa_Contig07261** | **5.7** | **3.2** | **75.3** | **41.9** | **13.0** | **4.5E-03** | **Hypothetical protein** |
| **Sa_Contig21041** | **16.7** | **7.3** | **214.7** | **92.4** | **12.7** | **3.0E-08** | **Tyrosine aminotransferase** |
| **Sa_Contig50581** | **37.3** | **40.8** | **547.7** | **510.5** | **12.5** | **2.2E-22** | **Hypothetical protein** |
| **Sa_Contig12742** | **84.3** | **24.4** | **1111.3** | **278.8** | **11.4** | **1.7E-44** | **Hypothetical protein** |
| **Sa_Contig15461** | **15.7** | **3.1** | **162.7** | **32.1** | **10.3** | **2.0E-05** | **Lichenase** |
| **Sa_Contig16808** | **12.0** | **1.6** | **86.0** | **14.4** | **9.2** | **3.7E-03** | **Mitochondrial chaperone BCS1-B** |
| **Sa_Contig10935** | **15.7** | **10.4** | **144.0** | **93.7** | **9.0** | **1.2E-04** | **Tyrosine aminotransferase** |
| **Sa_Contig14027** | **42.0** | **12.0** | **298.3** | **106.5** | **8.8** | **1.4E-11** | **Cationic peroxidase 2** |
| **Sa_Contig17598** | **19.7** | **7.7** | **137.0** | **67.9** | **8.8** | **3.8E-05** | **Peroxidase N1** |
| **Sa_Contig12361** | **17.3** | **5.5** | **135.3** | **46.9** | **8.6** | **1.7E-04** | **Transcription factor RAP2-3** |
| **Sa_Contig13861** | **29.7** | **3.9** | **190.7** | **25.8** | **6.7** | **1.2E-05** | **Trans-cinnamate 4-monooxygenase** |
| **Sa_Contig37386** | **28.0** | **9.2** | **163.0** | **60.3** | **6.6** | **1.6E-04** | **Hypothetical protein** |
| **Sa_Contig06552** | **28.0** | **2.4** | **170.7** | **14.1** | **5.9** | **2.1E-04** | **Metal-nicotianamine transporter YSL3** |
| **Sa_Contig15722** | **36.0** | **4.4** | **189.7** | **25.4** | **5.8** | **8.4E-05** | **4-coumarate--CoA ligase 1** |
| **Sa_Contig14529** | **42.7** | **24.0** | **210.7** | **135.4** | **5.6** | **1.8E-05** | **Metal transporter Nramp4** |
| **Sa_Contig04822** | **91.0** | **35.0** | **692.7** | **191.7** | **5.5** | **4.8E-15** | **Hypothetical protein** |
| **Contig Name** | **Cont** | | **Cd** | | **FC** | **q-value** | **Brief Description** |
|  | **reads** | **RPKM** | **reads** | **RPKM** |  |  |  |
| **Sa_Contig26445** | **31.7** | **4.8** | **137.3** | **24.6** | **5.2** | **3.2E-03** | **Flavanone 4-reductase** |
| **Sa_Contig25651** | **50.0** | **12.6** | **291.3** | **57.1** | **4.5** | **6.1E-05** | **membrane protein** |
| **Sa_Contig03765** | **39.0** | **20.1** | **166.0** | **90.8** | **4.5** | **2.8E-03** | **Metal transporter Nramp2** |
| **Sa_Contig29380** | **50.7** | **29.9** | **228.3** | **134.3** | **4.5** | **4.9E-05** | **Major allergen Pru av 1** |
| **Sa_Contig02608** | **53.7** | **14.1** | **201.3** | **63.1** | **4.5** | **2.1E-04** | **GDSL esterase/lipase** |
| **Sa_Contig04623** | **32.0** | **12.4** | **144.0** | **54.5** | **4.4** | **4.6E-03** | **Hypothetical protein** |
| **Sa_Contig30461** | **42.3** | **30.9** | **196.3** | **135.0** | **4.4** | **2.1E-04** | **Metal transporter Nramp3** |
| **Sa_Contig12913** | **50.3** | **11.1** | **178.3** | **43.7** | **3.9** | **2.7E-03** | **Cinnamyl alcohol dehydrogenase** |
| **Sa_Contig33556** | **235.7** | **175.0** | **734.0** | **689.6** | **3.9** | **5.9E-16** | **Hypothetical protein** |
| **Sa_Contig16769** | **68.0** | **9.8** | **272.3** | **38.5** | **3.9** | **7.6E-06** | **PQQ_DH domain-containing protein** |
| **Sa_Contig08963** | **189.0** | **19.6** | **671.7** | **76.0** | **3.9** | **2.2E-13** | **Oligopeptide transporter 3** |
| **Sa_Contig40025** | **57.0** | **17.4** | **170.3** | **64.9** | **3.7** | **3.5E-03** | **Protein phosphatase 2C 37** |
| **Sa_Contig12615** | **575.3** | **165.2** | **2381.7** | **612.0** | **3.7** | **4.7E-38** | **Hypothetical protein** |
| **Sa_Contig48217** | **130.0** | **47.4** | **381.0** | **171.3** | **3.6** | **3.8E-07** | **Hypothetical protein** |
| **Sa_Contig07242** | **201.3** | **113.9** | **652.3** | **411.4** | **3.6** | **2.0E-12** | **Hypothetical protein** |
| **Sa_Contig04922** | **235.0** | **155.3** | **878.0** | **559.0** | **3.6** | **2.2E-13** | **Hypothetical protein** |
| **Sa_Contig44473** | **123.3** | **73.2** | **341.0** | **253.1** | **3.5** | **4.5E-06** | **Hypothetical protein** |
| **Sa_Contig16498** | **137.3** | **108.1** | **381.3** | **373.6** | **3.5** | **4.3E-07** | **Hypothetical protein** |
| **Sa_Contig19227** | **61.7** | **31.7** | **190.0** | **103.7** | **3.3** | **4.5E-03** | **GDSL esterase/lipase** |
| **Sa_Contig10390** | **115.7** | **35.2** | **363.3** | **114.9** | **3.3** | **5.5E-06** | **Metal transporter Nramp3** |
| **Sa_Contig03766** | **127.0** | **25.9** | **354.0** | **81.0** | **3.1** | **1.4E-05** | **Purine permease 3** |
| **Sa_Contig01404** | **89.7** | **48.8** | **255.3** | **150.3** | **3.1** | **1.5E-03** | **3-ketoacyl-CoA synthase 1** |
| **Sa_Contig12923** | **96.7** | **17.9** | **252.3** | **54.8** | **3.1** | **1.1E-03** | **Phospho-2-dehydro-3-deoxyheptonate aldolase 1, chloroplastic** |
| **Sa_Contig39864** | **210.0** | **131.8** | **544.0** | **394.0** | **3.0** | **2.0E-08** | **Hypothetical protein** |
| **Sa_Contig11841** | **84.0** | **25.9** | **232.0** | **77.3** | **3.0** | **3.5E-03** | **Oligopeptide transporter 3** |
| **Sa_Contig12642** | **102.0** | **41.6** | **277.0** | **124.4** | **3.0** | **8.1E-04** | **Hypothetical protein** |
| **Sa_Contig12230** | **1141.0** | **455.0** | **3274.0** | **1337.1** | **2.9** | **1.2E-51** | **Hypothetical protein** |
| **Sa_Contig54698** | **152.3** | **114.9** | **416.0** | **334.9** | **2.9** | **2.5E-06** | **Hypothetical protein** |
| **Sa_Contig10182** | **87.0** | **24.6** | **302.0** | **71.2** | **2.9** | **8.6E-04** | **Hypothetical protein** |
| **Sa_Contig51707** | **98.0** | **41.1** | **246.7** | **117.2** | **2.8** | **3.6E-03** | **Hypothetical protein** |
| **Sa_Contig00140** | **100.0** | **38.9** | **260.3** | **110.1** | **2.8** | **2.8E-03** | **Hypothetical protein** |
| **Sa_Contig02729** | **131.7** | **59.5** | **308.0** | **160.9** | **2.7** | **1.3E-03** | **Probable ADP-ribosylation factor GTPase-activating protein AGD8** |
| **Sa_Contig05911** | **141.0** | **31.4** | **330.3** | **83.9** | **2.7** | **5.1E-04** | **Metalloendoproteinase 1** |
| **Sa_Contig04816** | **98.3** | **18.7** | **263.7** | **49.2** | **2.6** | **4.5E-03** | **Transcription factor RF2b** |
| **Sa_Contig09437** | **181.7** | **59.3** | **441.0** | **155.2** | **2.6** | **5.9E-05** | **Hypothetical protein** |
| **Sa_Contig02134** | **104.7** | **16.2** | **282.7** | **42.2** | **2.6** | **4.7E-03** | **Sucrose transport protein SUC2** |
| **Sa_Contig04378** | **183.0** | **15.6** | **427.0** | **40.5** | **2.6** | **1.3E-04** | **Phospho-2-dehydro-3-deoxyheptonate aldolase 1, chloroplastic** |
| **Sa_Contig10147** | **488.3** | **129.0** | **1279.3** | **332.0** | **2.6** | **1.3E-12** | **Hypothetical protein** |
| **Sa_Contig08243** | **150.3** | **27.2** | **326.7** | **69.0** | **2.5** | **1.1E-03** | **ABC transporter B family member 19** |
| **Contig Name** | **Cont** | | **Cd** | | **FC** | **q-value** | **Brief Description** |
|  | **reads** | **RPKM** | **reads** | **RPKM** |  |  |  |
| **Sa_Contig12379** | **741.0** | **268.3** | **1689.3** | **675.6** | **2.5** | **6.1E-21** | **Hypothetical protein** |
| **Sa_Contig09712** | **136.3** | **29.6** | **298.0** | **74.3** | **2.5** | **3.8E-03** | **Hypothetical protein** |
| **Sa_Contig00003** | **118.7** | **8.7** | **318.3** | **21.7** | **2.5** | **2.6E-03** | **Retrotransposon protein, putative, unclassified, expressed** |
| **Sa_Contig43609** | **218.3** | **44.9** | **464.0** | **111.4** | **2.5** | **5.2E-05** | **S-adenosylmethionine synthetase 2** |
| **Sa_Contig07003** | **176.0** | **24.3** | **376.0** | **59.8** | **2.5** | **9.4E-04** | **Methylenetetrahydrofolate reductase 2** |
| **Sa_Contig42088** | **372.0** | **283.9** | **791.3** | **694.2** | **2.4** | **1.9E-09** | **Peroxidase 42** |
| **Sa_Contig08370** | **170.3** | **39.5** | **376.7** | **96.4** | **2.4** | **5.7E-04** | **Ty3-gypsy retrotransposon protein** |
| **Sa_Contig09709** | **219.0** | **206.9** | **460.0** | **502.4** | **2.4** | **5.3E-05** | **Hypothetical protein** |
| **Sa_Contig22346** | **153.0** | **93.8** | **301.3** | **227.2** | **2.4** | **4.9E-03** | **NADH-ubiquinone oxidoreductase 20 kDa subunit, mitochondrial** |
| **Sa_Contig35492** | **224.7** | **153.8** | **532.0** | **371.9** | **2.4** | **4.5E-06** | **Hypothetical protein** |
| **Sa_Contig10693** | **152.0** | **33.6** | **442.3** | **81.1** | **2.4** | **1.6E-04** | **Metalloendoproteinase 1** |
| **Sa_Contig10201** | **623.0** | **220.1** | **1427.0** | **523.4** | **2.4** | **5.1E-14** | **Hypothetical protein** |
| **Sa_Contig00509** | **292.7** | **15.5** | **603.0** | **36.4** | **2.3** | **7.0E-06** | **ABC transporter B family member 19** |
| **Sa_Contig10380** | **495.7** | **207.7** | **1187.7** | **481.5** | **2.3** | **6.7E-13** | **Arabinogalactan peptide 20** |
| **Sa_Contig08259** | **1024.0** | **421.0** | **2031.7** | **953.9** | **2.3** | **1.0E-18** | **Hypothetical protein** |
| **Sa_Contig24276** | **500.3** | **460.3** | **1055.0** | **1031.2** | **2.2** | **1.9E-10** | **Hypothetical protein** |
| **Sa_Contig08300** | **198.7** | **156.3** | **424.3** | **347.9** | **2.2** | **4.7E-03** | **Hypothetical protein** |
| **Sa_Contig10427** | **280.3** | **45.4** | **529.0** | **99.3** | **2.2** | **1.6E-04** | **Trans-cinnamate 4-monooxygenase** |
| **Sa_Contig10508** | **1079.7** | **354.9** | **2450.3** | **773.2** | **2.2** | **3.7E-24** | **Xyloglucan endotransglucosylase/hydrolase** |
| **Sa_Contig12362** | **336.7** | **104.6** | **655.0** | **224.2** | **2.1** | **2.8E-05** | **Cellulose synthase-like protein D2** |
| **Sa_Contig09892** | **238.0** | **40.3** | **426.3** | **85.1** | **2.1** | **2.2E-03** | **Probable peptide transporter** |
| **Sa_Contig12236** | **402.7** | **67.9** | **695.0** | **142.1** | **2.1** | **1.4E-05** | **Hypothetical protein** |
| **Sa_Contig12226** | **223.3** | **48.9** | **454.0** | **101.4** | **2.1** | **1.9E-03** | **Heat stress transcription factor C-1** |
| **Sa_Contig11672** | **5657.3** | **2123.2** | **9937.3** | **4352.5** | **2.0** | **4.2E-88** | **Non-specific lipid-transfer protein** |
| **Sa_Contig09979** | **534.7** | **296.8** | **1117.3** | **604.9** | **2.0** | **5.4E-09** | **Xyloglucan endotransglucosylase/hydrolase** |
| **Sa_Contig11742** | **1934.0** | **474.4** | **4144.3** | **962.0** | **2.0** | **3.3E-36** | **Probable CCR4-associated factor 1 homolog 11** |
| **Sa_Contig12072** | **3035.7** | **1346.3** | **5808.3** | **2648.3** | **2.0** | **3.7E-45** | **Probable xyloglucan endotransglucosylase/hydrolase** |
| **Sa_Contig10818** | **565.3** | **188.7** | **1002.0** | **370.3** | **2.0** | **4.1E-07** | **Probable cellulose synthase A catalytic subunit 1** |
| **Sa_Contig10599** | **675.0** | **98.4** | **1336.7** | **193.1** | **2.0** | **5.2E-10** | **Transcription factor ERF9** |
| **Sa_Contig10631** | **255.7** | **49.6** | **472.7** | **97.2** | **2.0** | **4.3E-03** | **Glucomannan 4-beta-mannosyltransferase 2** |
| **Sa_Contig11163** | **277.7** | **87.7** | **519.7** | **171.4** | **2.0** | **4.0E-03** | **Probable methyltransferase PMT25** |
|  |  |  |  |  |  |  |  |
| **Sa_Contig12088** | **818.7** | **303.5** | **416.3** | **157.1** | **0.5** | **5.3E-05** | **E3 ubiquitin-protein ligase RGLG2** |
| **Sa_Contig26016** | **606.3** | **718.3** | **321.0** | **370.2** | **0.5** | **3.4E-04** | **Hypothetical protein** |
| **Contig Name** | **Cont** | | **Cd** | | **FC** | **q-value** | **Brief Description** |
|  | **reads** | **RPKM** | **reads** | **RPKM** |  |  |  |
| **Sa_Contig11520** | **1105.7** | **466.5** | **544.3** | **239.3** | **0.5** | **2.1E-07** | **Heat shock protein 81-3** |
| **Sa_Contig11408** | **925.3** | **293.3** | **459.0** | **149.8** | **0.5** | **5.9E-06** | **Coiled-coil domain-containing protein** |
| **Sa_Contig02110** | **752.7** | **110.7** | **374.0** | **56.1** | **0.5** | **4.8E-05** | **Retrotransposable element Tf2** |
| **Sa_Contig12639** | **2529.7** | **352.4** | **1187.0** | **176.1** | **0.5** | **1.4E-16** | **12-oxophytodienoate reductase 3** |
| **Sa_Contig48190** | **277.3** | **163.3** | **203.7** | **81.6** | **0.5** | **3.1E-03** | **Hypothetical protein** |
| **Sa_Contig11261** | **800.7** | **451.2** | **422.3** | **225.0** | **0.5** | **7.8E-06** | **Probable ADP-ribosylation factor GTPase-activating protein AGD13** |
| **Sa_Contig08207** | **764.7** | **101.2** | **363.3** | **50.4** | **0.5** | **2.5E-05** | **Phosphoenolpyruvate carboxylase 2** |
| **Sa_Contig10159** | **1226.7** | **234.5** | **560.7** | **116.8** | **0.5** | **2.1E-08** | **Hypothetical protein** |
| **Sa_Contig42339** | **609.0** | **490.3** | **258.7** | **243.3** | **0.5** | **1.2E-03** | **Hypothetical protein** |
| **Sa_Contig22110** | **351.7** | **397.7** | **223.7** | **194.3** | **0.5** | **1.6E-03** | **MLP-like protein 423** |
| **Sa_Contig39429** | **505.7** | **491.1** | **271.0** | **239.6** | **0.5** | **1.5E-03** | **Hypothetical protein** |
| **Sa_Contig51345** | **456.0** | **516.4** | **247.0** | **247.2** | **0.5** | **5.5E-04** | **Hypothetical protein** |
| **Sa_Contig42605** | **644.7** | **283.3** | **310.7** | **134.7** | **0.5** | **1.1E-04** | **Hypothetical protein** |
| **Sa_Contig17174** | **697.7** | **220.1** | **452.0** | **103.9** | **0.5** | **5.1E-09** | **Hypothetical protein** |
| **Sa_Contig49755** | **412.3** | **340.0** | **203.3** | **160.4** | **0.5** | **3.2E-03** | **Hypothetical protein** |
| **Sa_Contig33054** | **743.7** | **123.8** | **300.7** | **58.1** | **0.5** | **4.1E-05** | **membrane protein** |
| **Sa_Contig00577** | **3837.0** | **387.3** | **2621.7** | **180.9** | **0.5** | **1.3E-61** | **Tetratricopeptide repeat protein 27 homolog** |
| **Sa_Contig33862** | **679.7** | **357.0** | **317.7** | **166.5** | **0.5** | **1.4E-05** | **beta-fructofuranosidase, putative** |
| **Sa_Contig21688** | **557.3** | **305.9** | **371.3** | **142.0** | **0.5** | **6.3E-08** | **Hypothetical protein** |
| **Sa_Contig50103** | **946.0** | **846.2** | **428.3** | **392.7** | **0.5** | **4.3E-08** | **beta-amylase** |
| **Sa_Contig02652** | **918.3** | **551.0** | **381.0** | **253.9** | **0.5** | **2.0E-06** | **Hypothetical protein** |
| **Sa_Contig11740** | **945.3** | **245.5** | **381.7** | **113.1** | **0.5** | **3.5E-07** | **Cytokinin-O-glucosyltransferase 3** |
| **Sa_Contig39430** | **674.7** | **604.5** | **348.7** | **278.2** | **0.5** | **1.3E-06** | **Hypothetical protein** |
| **Sa_Contig25540** | **631.0** | **489.4** | **254.0** | **223.9** | **0.5** | **1.2E-04** | **Hypothetical protein** |
| **Sa_Contig22539** | **355.7** | **363.8** | **186.0** | **165.5** | **0.5** | **3.6E-03** | **Hypothetical protein** |
| **Sa_Contig39979** | **355.3** | **440.9** | **178.0** | **200.3** | **0.5** | **2.0E-03** | **Hypothetical protein** |
| **Sa_Contig21699** | **468.3** | **84.4** | **214.3** | **38.1** | **0.5** | **1.1E-03** | **DEAD-box ATP-dependent RNA helicase 51** |
| **Sa_Contig25629** | **434.3** | **73.1** | **169.7** | **32.8** | **0.4** | **2.7E-03** | **Ascorbate peroxidase, cytosolic** |
| **Sa_Contig10198** | **493.3** | **76.1** | **210.0** | **34.2** | **0.4** | **6.4E-04** | **MLO-like protein 8** |
| **Sa_Contig46796** | **419.3** | **359.6** | **220.7** | **161.2** | **0.4** | **5.4E-04** | **Hypothetical protein** |
| **Sa_Contig11423** | **336.7** | **116.8** | **166.7** | **52.3** | **0.4** | **3.1E-03** | **Auxin-induced protein 22B** |
| **Sa_Contig08086** | **563.7** | **42.6** | **217.0** | **18.8** | **0.4** | **2.3E-04** | **Tetraacyldisaccharide 4'-kinase** |
| **Sa_Contig11706** | **455.3** | **173.1** | **273.3** | **76.0** | **0.4** | **8.2E-06** | **MLP-like protein 43** |
| **Sa_Contig50741** | **290.0** | **211.8** | **175.0** | **92.6** | **0.4** | **1.7E-03** | **MLP-like protein 31** |
| **Sa_Contig45887** | **434.0** | **146.7** | **160.0** | **63.9** | **0.4** | **3.5E-03** | **Hypothetical protein** |
| **Sa_Contig11777** | **370.0** | **228.0** | **184.3** | **98.9** | **0.4** | **3.7E-04** | **Hypothetical protein** |
| **Sa_Contig01681** | **405.0** | **140.5** | **174.3** | **60.7** | **0.4** | **6.1E-04** | **Beta-galactosidase** |
| **Sa_Contig12888** | **443.7** | **151.1** | **214.7** | **65.0** | **0.4** | **6.0E-05** | **Chaperone protein dnaJ 8, chloroplastic** |
| **Sa_Contig17764** | **976.7** | **107.2** | **352.7** | **45.9** | **0.4** | **4.7E-09** | **Signal recognition particle** |
| **Contig Name** | **Cont** | | **Cd** | | **FC** | **q-value** | **Brief Description** |
|  | **reads** | **RPKM** | **reads** | **RPKM** |  |  |  |
| **Sa_Contig28745** | **451.3** | **248.2** | **208.0** | **105.6** | **0.4** | **2.4E-04** | **Hypothetical protein** |
| **Sa_Contig12334** | **456.7** | **205.4** | **222.3** | **87.2** | **0.4** | **3.1E-05** | **AP2/ERF and B3 domain-containing transcription repressor RAV2** |
| **Sa_Contig33272** | **824.0** | **288.5** | **313.3** | **122.4** | **0.4** | **1.5E-07** | **Cytokinin-O-glucosyltransferase 3** |
| **Sa_Contig11307** | **18914.0** | **13310.4** | **10324.7** | **5619.5** | **0.4** | **5.3E-280** | **No hits found** |
| **Sa_Contig06039** | **286.3** | **47.7** | **135.7** | **20.1** | **0.4** | **3.6E-03** | **BAH and TFIIS domain-containing protein** |
| **Sa_Contig09975** | **449.0** | **107.1** | **178.3** | **44.5** | **0.4** | **1.8E-04** | **Hypothetical protein** |
| **Sa_Contig10878** | **360.3** | **258.2** | **141.0** | **107.2** | **0.4** | **1.1E-03** | **Hypothetical protein** |
| **Sa_Contig07236** | **318.7** | **72.0** | **140.3** | **29.9** | **0.4** | **3.1E-03** | **Common plant regulatory factor 1** |
| **Sa_Contig00123** | **533.7** | **103.1** | **205.0** | **42.3** | **0.4** | **1.0E-04** | **Hypothetical protein** |
| **Sa_Contig10917** | **589.7** | **149.4** | **219.0** | **60.2** | **0.4** | **2.2E-05** | **Hypothetical protein** |
| **Sa_Contig10475** | **1507.0** | **177.1** | **587.3** | **71.3** | **0.4** | **2.0E-15** | **Glutamate dehydrogenase 2** |
| **Sa_Contig19201** | **337.7** | **65.9** | **129.3** | **26.2** | **0.4** | **2.1E-03** | **UPF0051 protein ABCI8, chloroplastic** |
| **Sa_Contig00963** | **241.7** | **130.0** | **108.3** | **51.4** | **0.4** | **3.8E-03** | **Probable protein phosphatase 2C 60** |
| **Sa_Contig12340** | **589.0** | **233.1** | **234.3** | **90.1** | **0.4** | **2.0E-06** | **Hypothetical protein** |
| **Sa_Contig28006** | **588.0** | **515.0** | **209.3** | **195.4** | **0.4** | **2.4E-07** | **Hypothetical protein** |
| **Sa_Contig52548** | **568.7** | **579.4** | **226.3** | **218.1** | **0.4** | **3.6E-07** | **Hypothetical protein** |
| **Sa_Contig01893** | **451.0** | **95.5** | **168.0** | **35.7** | **0.4** | **1.9E-05** | **Hypothetical protein** |
| **Sa_Contig00795** | **353.7** | **78.1** | **125.3** | **28.8** | **0.4** | **4.3E-04** | **UPF0051 protein ABCI8, chloroplastic** |
| **Sa_Contig08161** | **473.7** | **68.5** | **166.0** | **25.2** | **0.4** | **2.4E-05** | **4-hydroxyphenylacetaldehyde oxime monooxygenase** |
| **Sa_Contig11983** | **424.7** | **228.4** | **167.3** | **82.9** | **0.4** | **4.7E-06** | **Hypothetical protein** |
| **Sa_Contig33947** | **322.0** | **218.7** | **125.3** | **78.8** | **0.4** | **2.8E-04** | **Hypothetical protein** |
| **Sa_Contig06941** | **428.7** | **68.9** | **187.0** | **24.3** | **0.4** | **1.1E-06** | **Transcription factor IIIA** |
| **Sa_Contig03215** | **307.3** | **33.3** | **103.0** | **11.6** | **0.3** | **2.0E-03** | **Hypothetical protein** |
| **Sa_Contig04196** | **196.7** | **73.0** | **90.3** | **25.3** | **0.3** | **9.9E-04** | **Hydroxyproline-rich glycoprotein family protein** |
| **Sa_Contig46110** | **1582.7** | **804.0** | **566.3** | **275.4** | **0.3** | **9.6E-20** | **Hypothetical protein** |
| **Sa_Contig09643** | **642.7** | **171.8** | **237.7** | **58.5** | **0.3** | **1.2E-10** | **Phospholipase C 4** |
| **Sa_Contig18410** | **322.7** | **232.5** | **121.7** | **78.8** | **0.3** | **3.5E-05** | **Hypothetical protein** |
| **Sa_Contig12619** | **787.3** | **144.1** | **253.0** | **48.1** | **0.3** | **1.9E-10** | **Hypothetical protein** |
| **Sa_Contig22188** | **272.3** | **233.5** | **96.0** | **77.6** | **0.3** | **3.9E-04** | **17.6 kDa class I heat shock protein** |
| **Sa_Contig05162** | **249.7** | **161.2** | **82.7** | **53.6** | **0.3** | **1.2E-03** | **Hypothetical protein** |
| **Sa_Contig19919** | **272.0** | **51.7** | **83.3** | **17.1** | **0.3** | **9.4E-04** | **GDSL esterase/lipase** |
| **Sa_Contig23190** | **384.7** | **241.5** | **129.7** | **77.0** | **0.3** | **4.8E-06** | **Hypothetical protein** |
| **Sa_Contig11628** | **540.0** | **276.3** | **183.0** | **86.4** | **0.3** | **1.1E-09** | **Late embryogenesis abundant protein Lea5** |
| **Sa_Contig07287** | **343.3** | **206.4** | **131.7** | **64.2** | **0.3** | **2.1E-07** | **Hypothetical protein** |
| **Sa_Contig30868** | **1517.7** | **201.2** | **403.3** | **60.7** | **0.3** | **2.4E-22** | **Fyve finger-containing phosphoinositide kinase, fyv1, putative** |
| **Sa_Contig10356** | **333.3** | **173.4** | **111.7** | **51.8** | **0.3** | **3.1E-06** | **Hypothetical protein** |
| **Contig Name** | **Cont** | | **Cd** | | **FC** | **q-value** | **Brief Description** |
|  | **reads** | **RPKM** | **reads** | **RPKM** |  |  |  |
| **Sa_Contig07836** | **294.3** | **234.0** | **88.0** | **69.4** | **0.3** | **1.5E-04** | **Gibberellin 2-beta-dioxygenase** |
| **Sa_Contig09613** | **316.3** | **54.4** | **86.0** | **16.1** | **0.3** | **2.8E-04** | **1-aminocyclopropane-1-carboxylate oxidase homolog** |
| **Sa_Contig22439** | **801.3** | **648.3** | **280.3** | **189.4** | **0.3** | **3.9E-17** | **Hypothetical protein** |
| **Sa_Contig21345** | **186.0** | **74.8** | **56.0** | **21.5** | **0.3** | **2.7E-03** | **17.3 kDa class I heat shock protein** |
| **Sa_Contig26853** | **165.7** | **204.9** | **53.3** | **58.3** | **0.3** | **2.7E-03** | **Hypothetical protein** |
| **Sa_Contig53809** | **276.7** | **202.3** | **76.0** | **56.9** | **0.3** | **3.3E-04** | **Hypothetical protein** |
| **Sa_Contig02700** | **322.0** | **329.4** | **97.0** | **92.0** | **0.3** | **1.0E-05** | **Hypothetical protein** |
| **Sa_Contig15090** | **302.7** | **400.7** | **105.7** | **111.2** | **0.3** | **3.6E-07** | **Hypothetical protein** |
| **Sa_Contig38321** | **235.7** | **155.0** | **61.7** | **42.7** | **0.3** | **2.9E-03** | **Hypothetical protein** |
| **Sa_Contig13107** | **365.7** | **253.6** | **142.7** | **69.7** | **0.3** | **1.4E-10** | **Hypothetical protein** |
| **Sa_Contig09548** | **307.7** | **32.7** | **82.3** | **8.8** | **0.3** | **1.3E-04** | **Cytochrome P450 94A1** |
| **Sa_Contig31603** | **198.3** | **25.3** | **56.3** | **6.8** | **0.3** | **9.4E-04** | **Abscisic acid 8'-hydroxylase 3** |
| **Sa_Contig13098** | **1129.0** | **627.6** | **292.3** | **167.0** | **0.3** | **2.1E-23** | **Hypothetical protein** |
| **Sa_Contig03156** | **596.7** | **420.8** | **173.7** | **110.7** | **0.3** | **8.7E-13** | **Hypothetical protein** |
| **Sa_Contig47167** | **170.7** | **228.7** | **62.3** | **57.9** | **0.3** | **2.8E-05** | **Hypothetical protein** |
| **Sa_Contig09585** | **206.0** | **65.2** | **55.0** | **16.3** | **0.3** | **2.9E-04** | **HMG-box (high mobility group) DNA-binding family protein** |
| **Sa_Contig34329** | **162.7** | **192.3** | **40.3** | **47.6** | **0.2** | **1.2E-03** | **Hypothetical protein** |
| **Sa_Contig24559** | **250.0** | **130.9** | **59.3** | **32.3** | **0.2** | **4.3E-04** | **Peroxidase C2** |
| **Sa_Contig18339** | **271.0** | **108.7** | **65.3** | **25.8** | **0.2** | **2.5E-05** | **Hypothetical protein** |
| **Sa_Contig36109** | **265.0** | **219.9** | **56.7** | **49.6** | **0.2** | **1.2E-04** | **Cytochrome P450 94A1** |
| **Sa_Contig20529** | **97.3** | **163.1** | **30.3** | **36.6** | **0.2** | **3.3E-03** | **Hypothetical protein** |
| **Sa_Contig00593** | **117.0** | **174.2** | **35.0** | **38.9** | **0.2** | **2.5E-03** | **Hypothetical protein** |
| **Sa_Contig35723** | **150.0** | **80.0** | **34.0** | **17.4** | **0.2** | **2.0E-03** | **Hypothetical protein** |
| **Sa_Contig21040** | **272.3** | **336.5** | **73.7** | **71.5** | **0.2** | **1.8E-08** | **Hypothetical protein** |
| **Sa_Contig22105** | **123.0** | **130.1** | **32.0** | **26.1** | **0.2** | **6.2E-04** | **Hypothetical protein** |
| **Sa_Contig28480** | **615.3** | **511.0** | **101.7** | **96.1** | **0.2** | **1.3E-15** | **Metalloendoproteinase 1 precursor, putative** |
| **Sa_Contig05232** | **160.0** | **90.6** | **30.3** | **16.1** | **0.2** | **2.3E-04** | **Hypothetical protein** |
| **Sa_Contig24583** | **619.0** | **369.3** | **110.3** | **63.1** | **0.2** | **5.9E-18** | **Ferritin-3, chloroplastic** |
| **Sa_Contig30436** | **233.7** | **42.2** | **40.3** | **7.0** | **0.2** | **4.9E-07** | **Ethylene-responsive transcription factor ERF003** |
| **Sa_Contig36757** | **167.3** | **130.7** | **23.7** | **20.5** | **0.2** | **2.5E-04** | **Glucan endo-1,3-beta-glucosidase** |
| **Sa_Contig25918** | **154.3** | **128.8** | **24.7** | **20.1** | **0.2** | **2.1E-04** | **Hypothetical protein** |
| **Sa_Contig40801** | **394.3** | **284.4** | **46.3** | **42.5** | **0.1** | **1.1E-09** | **Pathogenesis-related protein 5** |
| **Sa_Contig27713** | **286.0** | **249.2** | **41.3** | **34.5** | **0.1** | **2.7E-09** | **Probable WRKY transcription factor 4** |
| **Sa_Contig04953** | **149.3** | **21.9** | **17.3** | **2.9** | **0.1** | **7.3E-04** | **Probable mannitol dehydrogenase** |
| **Sa_Contig45313** | **533.3** | **353.1** | **61.7** | **45.8** | **0.1** | **4.5E-16** | **Ferritin-3, chloroplastic** |
| **Sa_Contig04871** | **509.3** | **45.7** | **50.3** | **4.8** | **0.1** | **6.6E-18** | **Pyruvate kinase** |
| **Sa_Contig31362** | **270.0** | **31.2** | **27.0** | **3.2** | **0.1** | **2.6E-08** | **CBL-interacting serine/threonine-protein kinase 25** |
| **Contig Name** | **Cont** | | **Cd** | | **FC** | **q-value** | **Brief Description** |
|  | **reads** | **RPKM** | **reads** | **RPKM** |  |  |  |
| **Sa_Contig50518** | **94.0** | **93.8** | **9.0** | **9.3** | **0.1** | **4.0E-03** | **Hypothetical protein** |
| **Sa_Contig46287** | **83.7** | **24.9** | **9.3** | **2.5** | **0.1** | **3.5E-03** | **Hypothetical protein** |
| **Sa_Contig13539** | **542.3** | **265.4** | **50.0** | **26.1** | **0.1** | **3.8E-20** | **Ferritin-2, chloroplastic** |
| **Sa_Contig26521** | **200.3** | **105.8** | **16.7** | **9.1** | **0.1** | **9.2E-08** | **Hypothetical protein** |
| **Sa_Contig29588** | **192.0** | **140.7** | **19.7** | **11.9** | **0.1** | **6.7E-08** | **Hypothetical protein** |
| **Sa_Contig10364** | **2326.0** | **787.7** | **125.0** | **42.4** | **0.1** | **2.7E-112** | **Ferritin-2, chloroplastic** |
| **Sa_Contig13549** | **103.3** | **16.3** | **3.7** | **0.5** | **0.0** | **9.0E-04** | **Tyrosine N-monooxygenase** |
| **Sa_Contig56718** | **97.3** | **21.1** | **4.0** | **0.6** | **0.0** | **2.1E-06** | **Mannan endo-1,4-beta-mannosidase 4** |
| **Sa_Contig42555** | **704.7** | **214.9** | **16.3** | **5.1** | **0.0** | **7.8E-28** | **Uncharacterized membrane protein YCR023C** |

Notes: Expression abundance of each contig is the mean of three biological replicates and shown in RPKM (Reads Per Kilobase of exon model per Million total reads in sample). Fold Change (FC) = [Cd ]/[Cont].
