# Supplementary material for: Transcriptomic Analysis of Cadmium Stress Response in the Heavy Metal Hyperaccumulator Sedum alfredii Hance
Source: PLoS One. 2013 Jun 3;8(6):e64643. doi: 10.1371/journal.pone.0064643 (PMC3670878; doi:10.1371/journal.pone.0064643)
Supplement: Table S3 — List of PEPCs in S. alfredii Hance. (DOC) [file pone.0064643.s008.doc]

**Table S3 List of PEPCs in *S. alfredii* Hance**

| **Contig Name** | **Contig Length** | **Expression Abundance (RPKM)** | |
| --- | --- | --- | --- |
|  |  | **Cont** | **Cd** |
| **Sa_Contig00371** | **790** | **49.18** | **48.35** |
| **Sa_Contig05735** | **555** | **70.98** | **83.36** |
| **Sa_Contig06475** | **770** | **1.82** | **1.23** |
| **Sa_Contig08207** | **1648** | **101.15** | **50.44** |
| **Sa_Contig09998** | **655** | **85.47** | **39.96** |
| **Sa_Contig10983** | **345** | **238.63** | **224.44** |
| **Sa_Contig13496** | **230** | **0.51** | **0.61** |
| **Sa_Contig21128** | **239** | **31.56** | **34.86** |
| **Sa_Contig22700** | **758** | **44.32** | **51.07** |
| **Sa_Contig34860** | **221** | **209.31** | **192.33** |
| **Sa_Contig37511** | **577** | **1.53** | **0.76** |
| **Sa_Contig37512** | **449** | **1.13** | **1.11** |
| **Sa_Contig42658** | **360** | **0.66** | **1.88** |
| **Sa_Contig44508** | **3236** | **5.3** | **6.84** |
| **Sa_Contig49201** | **243** | **0.24** | **1.19** |
| **Sa_Contig49202** | **334** | **0.35** | **1.71** |
| **Sa_Contig50223** | **564** | **61.05** | **57.74** |
| **Sa_Contig50291** | **239** | **1.23** | **0.62** |
| **Sa_Contig52835** | **642** | **0.65** | **0.82** |
| **Sa_Contig55697** | **264** | **93.13** | **124.68** |
