# Supplementary material for: Transcriptomic Analysis of Cadmium Stress Response in the Heavy Metal Hyperaccumulator Sedum alfredii Hance
Source: PLoS One. 2013 Jun 3;8(6):e64643. doi: 10.1371/journal.pone.0064643 (PMC3670878; doi:10.1371/journal.pone.0064643)
Supplement: Table S4 — List of primers used for qRT-PCR. (DOC) [file pone.0064643.s009.doc]

**Table S4 List of primers used for qRT-PCR**

| **Contig Name** | **Brief Description** | **Forward Primer** | **Reverse Primer** |
| --- | --- | --- | --- |
| **Sa_Contig12607** | **1-Cys peroxiredoxin PER1** | **CTACACATCATAGGACCCGA** | **CGTCGGACAAACTTGGAGAT** |
| **Sa_Contig14694** | **Laccase1-1** | **GTCCTAATGTCTCTGATGCT** | **CTTCCACCACAGTCAGAGTG** |
| **Sa_Contig01582** | **Laccase 1-2** | **GTCCTAATGTCTCTGATGCT** | **CCTCAACCACTGTAAGAGTA** |
| **Sa_Contig13861** | **Cinnamate 4-hydroxylase** | **GAAGCTCAGCAGAAGGGAGA** | **GTTATTTGAACTCCTGGTCC** |
| **Sa_Contig12362** | **Cellulose synthase-like protein D2** | **GTACCATCTGCGGAACATTC** | **CGAGACACGTAGACTAGAAG** |
| **Sa_Contig43609** | **SAM synthetase 2** | **GTGTCTGATGATGTTGGTCT** | **GAGTCTCGTCAGTCGCGTAA** |
| **Sa_Contig03765** | **Metal transporter Nramp2** | **CTTCGCTACCCTTTTCATGGA** | **GCTACACCAAGTCTTGCTG** |
| **Sa_Contig30461** | **Metal transporter Nramp3** | **GCACTCATTTTCGACACTAG** | **CCGTTGATCACCATCACTAG** |
| **Sa_Contig11685** | **P-type metal ATPase HMA4** | **GATGCACCAGCACTAGCTAC** | **TCACCAACAAACACGTCCCA** |
| **Sa_Contig06552** | **Metal-nicotianamine transporter YSL3** | **TCAGACTTGCGCTGTTGCTT** | **CGTAAGGGTACTAAAGCTAG** |
| **Sa_Contig15461** | **Endo-1,3-beta-glucanase** | **GTCCTATAAACGGTGACACG** | **CGTTTCGAAACGAGCCTTGT** |
| **Sa_Contig09892** | **Probable peptide transporter** | **GGTGCTGATCAGTTGGACAA** | **GCTTGACGTACAATGGCGAT** |
| **Sa_Contig14529** | **Metal transporter Nramp4** | **CTCATTTGCTTGGATGTTTGG** | **TTCCCTTGACTGTACCAACG** |
| **Sa_Contig00501** | **Tyrosine aminotransferase** | **TGGGAGGCAAAGTTCGTTAC** | **GATTTCAATAGCCTGAGTGC** |
| **Sa_Contig10290** | **Zinc transporter ZIP1** | **GGAGCTGGGGATAGTAGTTC** | **GTAAGCGAGAAGAACAGCAC** |
| **Sa_Contig08243** | **ABC transporter B family member 19** | **ACCCAACGGCTACCAAACTC** | **GGTGGTTCGACCTCTCATGA** |
| **Sa_Contig08963** | **Oligopeptide transporter 3** | **TTCCGAGCGTTGCACGAGAA** | **GGCCTTTGTAACCTGATCCG** |
| **Sa_Contig47062** | **Metal tolerance protein MTP3** | **TCATCATAACCATGGCAGTG** | **GCATTAATCATCAGAGGCTC** |
| **Sa_Contig06989** | **Actin** | **TGTGCTTTCCCTCTATGCC** | **CGCTCAGCAGTGGTTGTG** |
