# Supplementary material for: Transcriptomic Analysis of Cadmium Stress Response in the Heavy Metal Hyperaccumulator Sedum alfredii Hance
Source: PLoS One. 2013 Jun 3;8(6):e64643. doi: 10.1371/journal.pone.0064643 (PMC3670878; doi:10.1371/journal.pone.0064643)
Supplement: Table S5 — Accession numbers of plant PEPCs for sequence alignment. (DOC) [file pone.0064643.s010.doc]

**Table S5 Accession numbers of plant PEPCs for sequence alignment**

| **Species** | **Sequence name** | **Accession number** |
| --- | --- | --- |
| **C3 plant** | | |
| ***Arabidopsis thaliana*** | **AtPEPC1_C3_type** | **At1g53310** |
| **C4 plants** | | |
| ***Zea mays*** | **ZmPEPC2.1_C3_type** | **CAA43709.1** |
| ***Zea mays*** | **ZmPEPC2.2 _C4_type** | **CAA27270.1** |
| ***Sorghum vulgare*** | **SvPEPC_C3_type** | **CAA42549.1** |
| ***Saccharum officinarum*** | **SvPEPC_C4_type** | **CAA35251.2** |
| ***Saccharum officinarum*** | **SoPEPC_C4_type** | **CAC08829** |
| **CAM plants** | | |
| ***Mesembryanthemum crystallinum*** | **McPEPC1_C3_type** | **CAA31956.1** |
| ***Mesembryanthemum crystallinum*** | **McPEPC2_CAM_type** | **P16097.1** |
| ***Kalanchoë blossfeldiana*** | **KbPEPC3_C3_type** | **CAA61085.1** |
| ***Kalanchoë blossfeldiana*** | **KbPEPC1_CAM_type** | **CAA61083.1** |
| ***Kalanchoë blossfeldiana*** | **KbPEPC2_CAM_type** | **CAA61084.1** |
| ***Clusia venosa*** | **CvPEPC2_CAM_type** | **DQ320117** |
| ***Clusia rosea*** | **CrPEPC2_CAM_type** | **DQ320119** |
| ***Clusia schomburgkiana*** | **CsPEPC2_CAM_type** | **DQ320112** |
| ***Clusia schomburgkiana*** | **CsPEPC3_CAM_type** | **DQ320113** |
| ***Clusia hilariana*** | **ChPEPC2_CAM_type** | **DQ320103** |
| ***Clusia aripoensis*** | **CaPEPC_CAM_type** | **DQ320115** |
